# Lenalidomide Plus Dexamethasone as FIRST‐Line Therapy in Transplant‐Ineligible Patients With Multiple Myeloma: Final Results of the Prospective, Non‐Interventional Study FIRST‐NIS and Comparison With the FIRST Pivotal Phase III Clinical Trial

**DOI:** 10.1002/cam4.71758

**Published:** 2026-04-02

**Authors:** H. Nückel, T. Behlendorf, H. Schulz, M. Schulze, C. Schardt, T. Medinger, M. Koenigsmann, T. Dechow, M. Indorf, D. Bürkle, V. Engelbertz, J. Rauh, B. Schmidt, A. Sauer, C. Vannier, K. Potthoff

**Affiliations:** ^1^ Hämatologisch/Onkologische Gemeinschaftspraxis Prof. Dr. Nückel/Dr. Matschke Bochum Germany; ^2^ MVZ III Onkologie der Evidia MVZ Halle (Saale) GmbH Halle (Saale) Germany; ^3^ Praxis Internistischer Onkologie und Hämatologie (PIOH) Frechen Germany; ^4^ Praxis und Tagesklinik für Hämatologie/Onkologie Zittau Germany; ^5^ Gemeinschaftspraxis Dres. Schardt & Azeh Gelsenkirchen Germany; ^6^ iOMEDICO Freiburg Germany; ^7^ MediProjekt GbR Hannover Germany; ^8^ Studienzentrum Onkologie Ravensburg Ravensburg Germany; ^9^ Zentrum für Ambulante Onkologie Schorndorf Germany; ^10^ MVZ Onkologie an der St. Marien‐Hospital gGmbH Düren Germany; ^11^ GIM Gemeinschaftspraxis für Innere Medizin, Studien‐ und Fortbildungs GbR Witten Germany; ^12^ Hämatologie und Onkologie München Pasing MVZ GmbH Munich Germany; ^13^ BAG Onkologie am Filmpark Potsdam Germany

**Keywords:** dexamethasone, Germany, lenalidomide, multiple myeloma, non‐interventional study, routine clinical practice, transplant‐ineligible patients

## Abstract

**Objectives:**

Lenalidomide and low‐dose dexamethasone (Rd) is a standard regimen for transplant‐ineligible (TIE) patients with newly diagnosed multiple myeloma (NDMM), for whom a multi‐drug treatment regimen is not considered appropriate. While Rd has been intensively investigated in clinical trials, prospective real‐world data are still scarce.

**Patients and Methods:**

The prospective, multicenter, non‐interventional study FIRST‐NIS (NTC02537808) investigated the effectiveness, safety, and quality of life (QoL) of Rd in a German real‐world setting. G8‐Geriatric assessment was used to assess patients' impairment status. Patients were treated according to the physician's discretion. Data were analyzed descriptively; no formal hypothesis was tested.

**Results:**

Between 2015 and 2018, 168 patients with TIE NDMM were included, median age was 77.7 years. With a median follow‐up of 64.2 months, median progression‐free survival (PFS) in the real‐world setting was 22.9 months [95% CI 19.3, 28.1], median overall survival (OS) 58.1 months [95% CI 45.7, 71.7]. Patients ≤ 75 years and non‐impaired patients showed a more favorable PFS and OS, and Rd was a feasible treatment option in most patients with renal impairment. QoL was maintained during Rd treatment. No new safety signals emerged.

**Conclusions:**

The results of the FIRST‐NIS support Rd as an effective and safe frontline treatment option for patients with TIE NDMM, irrespective of age, with similar clinical outcomes in the real world compared to the pivotal trial.

**Trial Registration:**

ClinicalTrials.gov identifier: NCT02537808

## 1 Introduction

Lenalidomide and low‐dose dexamethasone (Rd) is an established standard of care frontline regimen for newly diagnosed multiple myeloma (NDMM) patients for whom a multi‐drug treatment regimen is not considered appropriate and who are not eligible for autologous transplantation. In Europe, authorization of Rd was granted in 2015 based on the results of the pivotal phase III FIRST trial showing a significantly prolonged progression‐free survival (PFS) and overall survival (OS) in patients of all ages treated with either continuous Rd until progression (Rdc) or Rd for 18 cycles (Rd18) compared with melphalan‐prednisone‐thalidomide (MPT) [1, 2].

About one‐third of the transplant‐ineligible (TIE) patients with NDMM are older than 75 years at diagnosis. This group of patients is highly heterogeneous, exhibiting marked variation in their general state of health and disease characteristics, such as comorbidities and functional impairments [3]. However, age per se does not necessarily capture frailty, which is an age‐associated state of cumulative decline in many physiological systems [4, 5, 6]. Thus, over the past decade, geriatric and comorbidity assessment tools have been developed to further evaluate the suitability of treatment regimens and prognosis in this patient population [7, 8, 9, 10].

In the FIRST trial, 35% of the patients were older than 75 years at diagnosis [1], and shorter median PFS and OS were reported for Rd in patients aged > 75 years (Rdc: 20.3/48.3 months; Rd18: 19.4/45.7 months) compared to the patients ≤ 75 years (Rdc: 28.1/66.9 months; Rd18: 21.6/71.5 months) [2, 11]. Moreover, Facon et al. developed a simplified frailty scale based on age, Eastern Cooperative Oncology Group (ECOG) performance status (PS) and Charlson Comorbidity Index (CCI). Using the data from the FIRST trial, it was shown that frail patients treated with Rd had worse PFS and OS (19.4/42.1 months) compared to non‐frail patients (24.0/70.1 months), demonstrating the prognostic value of the frailty score [10].

Among functional impairments, renal impairment (RI) is one of the most common complications of multiple myeloma (MM) which requires cautious therapy management. About half of the patients in the FIRST trial presented with RI at baseline, and Rd treatment, with renally adapted R dosing, was shown to be effective for most patients with RI [1, 12]. Although bortezomib‐based therapy regimens are the cornerstone of the management of MM‐related RI, Rd is considered an effective and safe therapy option in patients mainly with mild to moderated RI [13].

Given that MM is an incurable disease and patients experience a variety of symptoms during treatment, maintaining health‐related quality of life (HRQoL) by fine‐tuning anti‐myeloma therapy is an important treatment goal [14]. In the FIRST trial, treatment with Rd was associated with a clinically meaningful improvement in HRQoL, as measured using validated questionnaires (QLQ‐C30, QLQ‐MY20, and EQ‐5D) [15].

Since 2019, the treatment landscape of MM has been changing substantially, as the combination of bortezomib with Rd (VRd) and the addition of daratumumab to VMP (DaraVMP) or Rd (DaraRd) had become new frontline standards of care [16]. In the randomized phase III MAIA trial, at a median follow‐up of 56.2 months a significant benefit in OS was observed in the DaraRd group compared to Rd alone (hazard ratio [HR] 0.68; 95% CI 0.53–0.86; *p* = 0.0013) [17] and median PFS was 52.2 versus 34.4 months in the Rd control group [18]. Moreover, based on the results of the IMROZ phase III trial, the combination of isatuximab and VRd (Isa‐VRd) will soon be available as another effective treatment option [19]. However, these multi‐drug treatment regimens are linked to a higher incidence of adverse events (AE) when compared to established regimens like Rd alone [17, 19, 20, 21, 22]. Thus, Rd is still recommended as an effective and safe therapy option in patients who cannot receive these therapy regimens [16]. However, selecting the appropriate treatment strategy for very elderly and frail patients with NDMM remains a challenge for the treating physicians.

Prospective real‐world data of this commonly used regimen, especially in patients > 75 years and accompanying evaluation of patient‐reported HRQoL, are still scarce. Moreover, there is a paucity of data pertaining to the utilization of the G8‐Geriatric assessment (G8‐GA) tool in patients with TIE NDMM. The FIRST‐NIS was designed and aimed to prospectively evaluate the effectiveness, safety and patient‐reported HRQoL of Rd treatment in Germany in a real‐world setting.

## 2 Methods

### 2.1 Study Design and Setting

FIRST‐NIS (NTC02537808) was a prospective, multicenter, non‐interventional study (NIS). The study was approved by the responsible ethics committees and conducted in accordance with the national regulations and the principles of the Declaration of Helsinki. Written informed consent was obtained from all patients before study enrolment. Patients were recruited in haemato‐oncological sites, including hospitals, outpatient clinics, and independent practices, across Germany from June 2015 through July 2018. Long‐term follow‐up ended in September 2023.

### 2.2 Patients and Treatment

Eligible patients were aged ≥ 18 years with confirmed diagnosis of TIE NDMM and decision for first‐line combination therapy with Rd in accordance with the treatment guidelines provided in summary of product characteristics (SmPC) [23]. As per SmPC, lenalidomide is administered at a recommended dosage of 25 mg once daily on days 1–21 of 28‐day cycles; recommended dexamethasone dosage is 40 mg on days 1, 8, 15, and 22 of each 28‐day cycle. Lenalidomide starting dose could be reduced according to the severity of renal impairment, and a starting dose of 20 mg dexamethasone is recommended for patients aged ≥ 75 years.

The decision to treat the patient with Rd was at the discretion of the treating physician and independent of the decision to enroll the patient into the NIS. Due to the teratogenicity of lenalidomide, which can cause severe birth defects if taken during pregnancy, patients were required to comply with the requirements of the pregnancy prevention program as part of the clinical routine in order to be eligible for Rd treatment [23]. A comprehensive documentation of patient and disease characteristics including G8‐GA [24] was conducted at baseline [25]. Rd therapy was documented in detail up to a maximum of 24 months treatment observation period (TOP) or until disease progression (PD), death or any other reason for treatment discontinuation according to the discretion of the treating physician. If applicable, the patients were followed up for the last day of lenalidomide administration and the further course of disease, OS, and subsequent line of treatment up to 36 months after the end of TOP of the last patient.

### 2.3 Study Objectives

The primary objective of the NIS was to assess the 24‐month PFS‐rate, defined as the proportion of patients who were alive and without PD at 24 months after start of Rd therapy. PD was assessed according to the International Myeloma Working Group (IMWG) response criteria [26], based on the treating physician's assessment (overall response) in routine clinical practice.

Secondary effectiveness objectives included the overall response rate (ORR) (according to the IMWG response criteria for MM [26]), PFS, time to response (TTR), duration of response (DOR), time to second‐line anti‐myeloma therapy (TT2nd‐line AMT), and OS. Further secondary objectives included patient‐reported HRQoL and treatment‐emergent AEs.

### 2.4 Adverse Events

Treatment‐emergent AEs were recorded from the day of first administration of study treatment until 30 days of the last dose of lenalidomide administration or the end of the 24‐month TOP, whatever came first. AEs were graded according to the National Cancer Institute Common Terminology Criteria for AEs (NCI CTCAE version 4.03) [27] and classified to preferred terms according to the Medical Dictionary for Regulatory Activities (MedDRA version 26.0). AE of specific interest were pre‐defined based on the known lenalidomide safety profile [23]. Second primary malignancies (SPM) were documented from the time of signed informed consent until end of study for each patient.

### 2.5 Health‐Related Quality of Life

The European Organization for Research and Treatment of Cancer (EORTC) QLQ‐C30 and EORTC QLQ‐MY20 questionnaires were employed to evaluate patients' HRQoL during the TOP [28, 29]. The analysis was focused on six clinically relevant HRQoL domains as previously reported for the pivotal trial (QLQ‐C30: Global health status (GHS), physical functioning, fatigue, pain; QLQ‐MY20: Disease symptoms, side effects of treatment) [15]. Patients were requested to complete the questionnaires at baseline and 3, 6, 12, 18, and 24 months after the start of Rd treatment. Changes in HRQoL were considered clinically relevant according to the established definition of minimal important differences (MID) for EORTC QLQ‐C30 and EORTC‐MY20 [30, 31].

### 2.6 Statistical Analysis

The full analysis set (FAS) comprised all eligible patients who had received at least one dose of lenalidomide in accordance with the SmPC [23]. This set was employed to assess patient and disease characteristics, effectiveness (including pre‐defined subgroups according to patient baseline data), and QoL. The safety analysis set (SAF) consisted of all patients who had received at least one dose of lenalidomide and for whom at least one post‐baseline information under treatment was available. The SAF was the relevant population for the evaluation of safety (AE analysis), exposure and treatment data. All variables were analyzed descriptively. Nominal variables were analyzed using frequencies and percentages (with 95% confidence intervals (CI) for percentages, where applicable); continuous variables were analyzed using the number of observations (*N*), the mean, the 95%‐CI for the mean, the standard deviation, the median, the minimum and the maximum. Time‐to‐event data were analyzed using the median and quartiles according to the Kaplan–Meier method [32], including the respective 95% CIs [33], as well as the frequency and percentage of censored cases. The specific time‐to‐event variables in this trial were PFS, TTR, TT2nd‐line AMT, OS, all calculated as the time from start of lenalidomide therapy to the date of documented event, and DOR, calculated as the time from first documented response [≥ partial response (PR)] to the date of documented event. Predefined subgroups were determined based on their established clinical relevance in multiple myeloma and the availability of comparable subgroup analyses in the pivotal FIRST trial. Specifically, subgroups were defined by age (≤ 75 vs. > 75 years [2, 11]), renal function (based on creatinine clearance [1, 12]), and impairment status (using the G8‐Geriatric Assessment tool, as recommended at the time of study conception by the EORTC in the Elderly Task Force [7, 24, 25]), as these baseline factors are known to influence prognosis and treatment outcomes in transplant‐ineligible NDMM patients. All subgroup analyses were considered exploratory. The follow‐up time was displayed with descriptive statistics for surviving patients only. A multivariate Cox regression analysis was performed to identify pre‐defined potential baseline factors, which might have an impact on PFS [34]. Further details on the methodology are provided in the supporting appendix (Table S6). All statistical analyses were calculated using SAS Version 9.4 of the SAS System for Windows. Copyright 2002–2012 SAS Institute Inc. SAS and all other SAS Institute Inc. product or service names are registered trademarks or trademarks of SAS Institute Inc., Cary, NC, USA.

## 3 Results

The objective of the FIRST‐NIS was to generate real‐world data on frontline Rd treatment in patients with TIE NDMM and to compare the results with the pivotal trial. Therefore, a descriptive, exploratory comparison of the most important data is presented in the supporting appendix (Table S1–S4).

### 3.1 Patient and Disease Characteristics at Baseline

**FIGURE 1:**
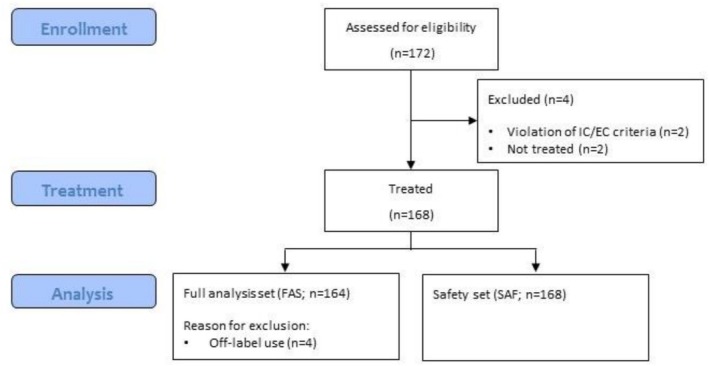
Consort diagram. Reason for exclusion from full analysis set (FAS): Four patients were excluded from the analysis population as they started the anti‐myeloma therapy with bortezomib and therefore were not treated according to the summary of product characteristics (off‐label use). These patients remained in the safety analysis set of the study (SAF). EC, exclusion criteria; FAS, full analysis set; IC, inclusion criteria.

Between June 2015 and July 2018, 168 patients treated with Rd were enrolled by 41 sites. In total, 164 patients were included in the FAS and 168 patients were eligible for the SAF (Figure 1). The main characteristics of the patients at baseline in the FAS and stratified by age (> 75 vs. ≤ 75 years) are summarized in Table 1.

**TABLE 1:** Patient demographics and clinical characteristics at baseline (FAS) and stratified by age (> 75 vs. ≤ 75 years).

	FAS (*N* = 164)	> 75 years (*N* = 116)	≤ 75 years (*N* = 48)
Patient characteristics			
Age at inclusion (years)			
Median, (range)	77.7 (53.6, 87.4)	79.3 (75.1, 91.3)	71.8 (53.6, 74.9)
Sex [*n*, (%)]			
Female	91 (55.5)	69 (59.5)	22 (45.8)
Male	73 (44.5)	47 (40.5)	26 (54.2)
ECOG PS [*n*, (%)]			
0/1	126 (76.8)	87 (75.0)	39 (81.3)
≥ 2	29 (17.7)	22 (19.0)	7 (14.6)
Missing	9 (5.5)	7 (6.0)	2 (4.2)
G8‐Geriatric assessment score^a^ [*n*, (%)]			
> 14	33 (20.1)	23 (19.8)	10 (20.8)
≤ 14	86 (52.4)	65 (56.0)	21 (43.8)
Unknown/missing	45 (27.4)	28 (24.1)	17 (35.4)
Comorbidities (any) [*n*, (%)]			
0/1	29 (17.7)	21 (18.1)	8 (16.7)
≥ 2	135 (82.3)	95 (81.9)	40 (83.3)
ISS stage [*n*, (%)]			
Stage I	38 (23.2)	24 (20.7)	14 (29.2)
Stage II	60 (36.6)	37 (31.9)	23 (47.9)
Stage III	40 (24.4)	32 (27.6)	8 (16.7)
Unknown/missing	26 (15.9)	23 (19.8)	3 (6.3)
Myeloma subtype—Immune globulins and light chains [*n*, (%)]			
IgA	26 (15.9)	21 (18.1)	5 (10.4)
IgA and IgG	2 (1.2)	1 (0.9)	1 (2.1)
IgG	100 (61.0)	74 (63.8)	26 (54.2)
IgM	1 (0.6)	0 (0.0)	1 (2.1)
Light chain only	30 (18.3)	17 (14.7)	13 (27.1)
Other	5 (3.0)	3 (2.6)	2 (4.2)
CrCl (mL/min) [*n*, (%)]			
< 30	16 (9.8)	14 (12.1)	2 (4.2)
30–< 60	71 (43.3)	61 (52.6)	10 (20.8)
60–< 90	60 (36.6)	37 (31.9)	23 (47.9)
≥ 90	17 (10.4)	4 (3.4)	13 (27.1)
Cytogenetic risk profile^b^ [*n*/total *n*, (%)]			
Standard risk	97/109 (89.0)	69/78 (88.5)	28/31 (90.3)
High risk	12/109 (11.0)	9/78 (11.5)	3/31 (9.7)

Abbreviations: CrCl, creatinine clearance; ECOG PS, eastern cooperative oncology group performance status; FAS, full analysis set; Ig, immunoglobulin; ISS, international staging system.

^a^ G8‐Geriatric assessment score: G8‐GA score of > 14 indicates “non‐impaired”, ≤ 14 indicates “impaired” patient status.

^b^ Definition according to pivotal trial: At least a translocation *t*(4;14), or *t*(14;16), or deletion del(17p). Test results were available for 109/164 (66.5%) patients.

The median age was 77.7 years, with most patients (*n* = 116; 70.7%) older than 75 years. An Eastern Cooperative Oncology (ECOG) status of 0 or 1 was documented in 76.8% of the patients. A total of 135 patients (82.3%) presented with at least two comorbidities. Most patients had an International Staging System (ISS) stage I or II (59.8%). The cytogenetic risk profile was available for 109 patients (66.5%), with 12/109 patients (11.0%) classified into high‐risk and 97/109 (89.0%) into standard‐risk.

The median age was 79.3 years for the subgroup of patients > 75 years and 71.8 years for patients ≤ 75 years, the frequency of patients documented with ≥ 2 comorbidities was similar (81.9% vs. 83.3%). Both the proportion of patients with documented ISS stage III (27.6% vs. 16.7%) and with renal impairment defined as creatinine clearance (CrCl) < 60 mL/min (64.7% vs. 25.0%, respectively) were elevated in the subgroup of patients > 75 years.

Based on the G8‐GA [25, 35], 86 patients (52.4%) were considered impaired (G8‐GA score ≤ 14) versus 33 patients (20.1%) who were classified as non‐impaired (G8‐GA score > 14). While the median age was similar (78.8 years vs. 76.4 years), more impaired patients presented with ≥ 2 comorbidities (89.5% vs. 66.7%) and an ECOG PS ≥ 2 (23.2% vs. 0.0%). Both the proportion of patients with documented ISS stage III (31.4% vs. 24.2%) and with RI (defined as CrCl < 60 mL/min) were elevated in the subgroup of impaired patients (62.8% vs. 33.3%, respectively). An overview of selected patient and disease characteristics stratified by age and G8‐GA score is available in the supporting appendix (Table S13).

### 3.2 Treatment With Lenalidomide and Dexamethasone

Rd treatment characteristics and stratified by age are summarized in Table 2. A lenalidomide starting dose of 25 mg was documented in 92 patients (54.8%); 76 patients (45.2%) received starting doses < 25 mg. The median duration of lenalidomide treatment was 7.6 months (95% CI [6.3, 9.9]), with a median lenalidomide dose intensity of 16.5 mg (range [4.3, 25.0]) per day of administration.

**TABLE 2:** Lenalidomide and dexamethasone treatment details [SAF/stratified by age (> 75 vs. ≤ 75 years)].

Treatment details	SAF (*N* = 168)	> 75 years (*N* = 116)	≤ 75 years (*N* = 52)
Lenalidomide			
Starting dose [*n*, %]			
25 mg	92 (54.8)	56 (48.3)	36 (69.2)
< 25 mg	76 (45.2)	60 (51.7)	16 (30.8)
20 mg	19 (11.3)	13 (11.2)	6 (11.5)
15 mg	33 (19.6)	26 (22.4)	7 (13.5)
10 mg	18 (10.7)	16 (13.8)	2 (3.8)
< 10 mg	6 (3.6)	5 (4.3)	1 (1.9)
Treatment duration (months)			
Median (95% CI)	7.6 (6.3, 9.9)	6.9 (5.0, 8.0)	14.9 (7.1, 24.9)
Number of cycles (*n*)			
Median (range)	8.0 (1, 26)	6.0 (1, 26)	12.0 (1, 26)
Mean (StD)	11.0 (9.0)	9.6 (8.2)	14.0 (9.9)
Dose/day of administration^a^ (mg)			
Median (min–max)	16.5 (4.3, 25.0)	15.0 (4.3, 25.0)	18.9 (7.5, 25.0)
Dexamethasone^b^			
Starting dose [*n*, %]			
40 mg	39 (23.2)	16 (13.8)	23 (44.2)
20 mg	109 (64.9)	86 (74.1)	23 (44.2)
< 20 mg	20 (11.9)	14 (12.1)	6 (11.6)
Number of cycles (n)			
Median (min‐max)	7.0 (1, 26)	6.0 (1, 26)	9.0 (1, 26)
Mean (StD)	10.0 (8.4)	9.1 (7.9)	11.9 (9.3)
2nd‐line anti‐myeloma therapy [*n*, (%)]			
At least one 2nd‐line AMTc	94 (57.3)	n.a.	n.a.
Bortezomib‐based regimen	57/94 (60.6)	n.a.	n.a.
Lenalidomide‐based regimen	13/94 (13.8)	n.a.	n.a.

Abbreviations: AMT, anti‐myeloma therapy; CI, confidence interval; Max, maximum; Mg, milligram; Min, minimum; N.A., not analyzed; *N*/*n*, number; SAF, safety analysis set; SD, standard deviation.

^a^ Lenalidomide daily dose was defined as the cumulative dose divided by dose exposure (mg/day).

^b^ As per statistical analysis plan, dexamethasone treatment details were not further analyzed. Category 20 mg includes two patients (> 75 years) documented with a dexamethasone starting dose of 24 mg.

Dexamethasone was given at a starting dose of 20 mg in 109 patients (64.9%), while 39 patients (23.2%) received a starting dose of 40 mg. Median number of dexamethasone treatment cycles was 7.0 (range 1–26).

In the subgroup of patients > 75 years, 56 patients (48.3%) were started on a lenalidomide dose of 25 mg versus 36 patients (69.2%) in patients ≤ 75 years. The median duration of lenalidomide treatment in patients > 75 years was 6.9 months (95% CI 5.0, 8.0) versus 14.9 months (95% CI 7.1, 24.9) in patients ≤ 75 years. The median lenalidomide dose intensities were 15.0 versus 18.9 mg per day of administration. Dexamethasone starting doses of ≤ 20 mg (*n* = 100; 86.2%) were reported in the majority of patients > 75 years, whereas a starting dose of 40 mg was documented in 23 patients (44.2%) aged ≤ 75 years.

Based on the G8‐GA, the median duration of lenalidomide treatment was 6.8 months (95% CI 4.6, 8.1) in the subgroup of impaired patients versus 9.9 months (95% CI 3.8, 22.0) in non‐impaired patients. Corresponding median lenalidomide dose intensities were 15.3 versus 20.7 mg per day of administration. Lenalidomide starting doses and further treatment characteristics based on G8‐GA and in the subgroups of patients stratified by CrCl at baseline are available in the supporting appendix (Tables S13 and S5, respectively).

### 3.3 Effectiveness

Survival and response to therapy in the total patient population.

**FIGURE 2:**
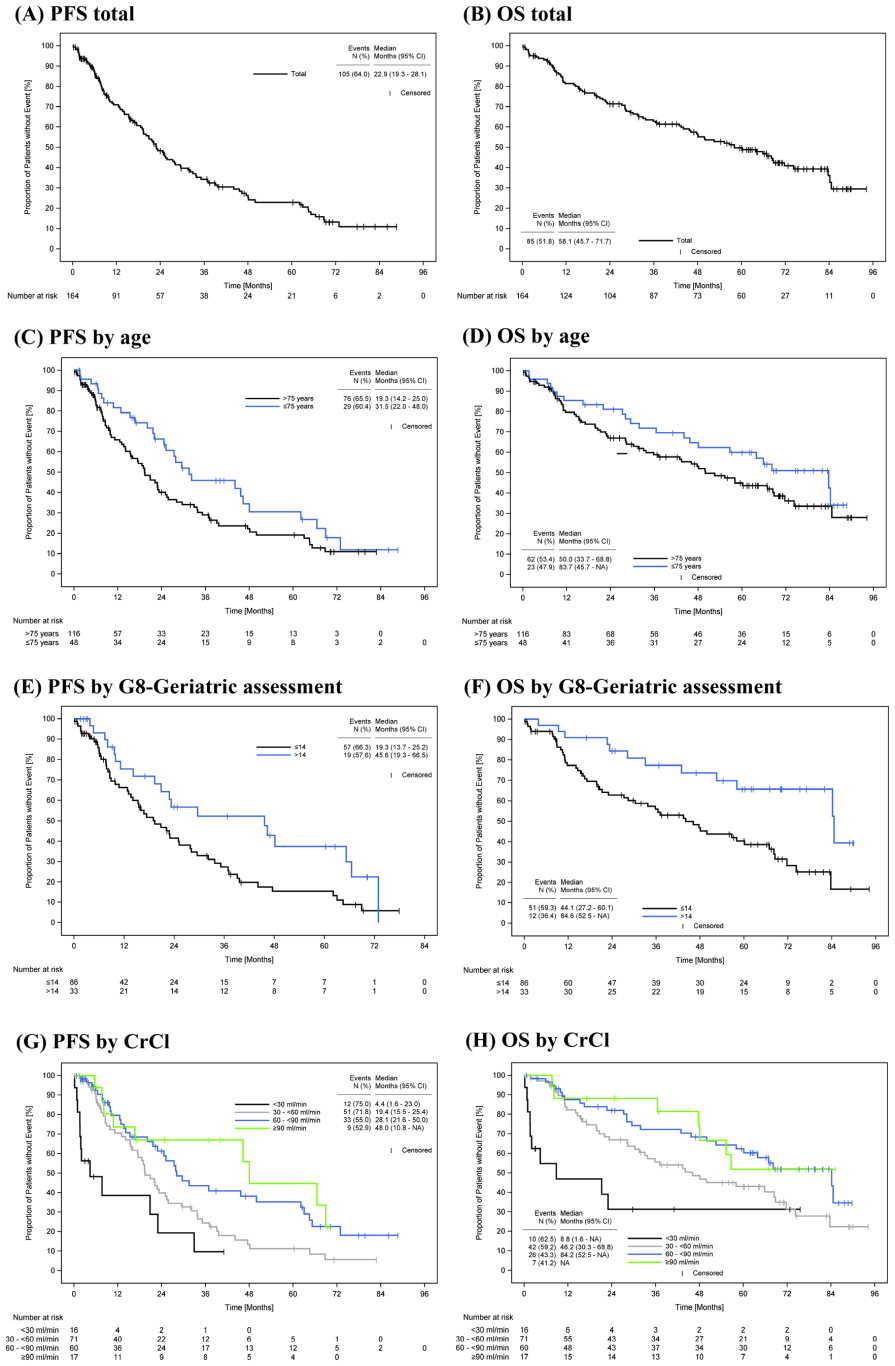
Kaplan–Meier estimates of progression‐free survival and overall survival (A, B) in the total population (FAS), (C, D) stratified by age (> 75 years vs. ≤ 75 years), (E, F) stratified by G8‐Geriatric assessment score, and (G, H) stratified by estimated CrCl (mL/min), documented at baseline. CI, confidence interval; CrCl, creatinine clearance; FAS, full analysis set; OS, overall survival; PFS, progression‐free survival.

At the time of final database lock (September 2023) with a median follow‐up of 64.2 months, the 24‐month PFS‐rate was 48.3% (95% CI 39.4, 56.6) and the ORR was 59.1% (95% CI 51.5, 66.4) (Table 3). A total of 31 (18.9%) patients achieved a stringent CR (sCR) or very good partial remission (VGPR). Median TTR was 3.1 months (95% CI 2.5, 3.8) and median DOR was 28.4 months (95% CI 21.6, 42.9). Median PFS and OS were 22.9 months (95% CI 19.3, 28.1) and 58.1 months (95% CI 45.7, 71.7) (Table 3, Figure 2), respectively. A total of 94 (57.3%) patients received 2nd‐line AMT with a median TT2nd‐AMT of 29.7 months (95% CI 23.2, 37.5). Most frequently documented 2nd‐line treatments were bortezomib‐based (*n* = 57/94; 60.6%) and lenalidomide‐based (*n* = 13/94; 13.8%) therapy regimens (Table 2).

**TABLE 3:** Effectiveness (FAS) and stratified by age (> 75 vs. ≤ 75 years).

Variable	Total population (*N* = 164)	> 75 years (*N* = 116)	≤ 75 years (*N* = 48)
24‐month PFS‐rate			
24‐month rate (%)	48.3	40.1	66.3
(95% CI)	(39.4, 56.6)	(29.9, 50.1)	(49.6, 78.5)
Events [*n*, (%)]	105 (64.0)	76 (65.5)	29 (60.4)
Overall response rate^a^ [*n*, (%)]	97 (59.1)	63 (54.3)	34 (70.8)
(95% CI)	(51.5, 66.4)	(45.3, 63.1)	(56.7, 81.8)
Best response^b^ [*n*, (%)]			
Stringent complete response (sCR)	2 (1.2)	2 (1.7)	0 (0.0)
Very good partial response (VGPR)	29 (17.7)	18 (15.5)	11 (22.9)
Partial response (PR)	66 (40.2)	43 (37.1)	23 (47.9)
Stable disease (SD)	43 (26.2)	34 (29.3)	9 (18.8)
Progressive disease (PD)	3 (1.8)	3 (2.6)	0 (0.0)
Missing	21 (12.8)	16 (13.8)	5 (10.4)
Time to response (months)			
Median (95% CI)	3.1 (2.5, 3.8)	3.2 (2.5, 5.8)	2.8 (1.7, 3.6)
Events [*n*, (%)]	97 (59.1)	63 (54.3)	34 (70.8)
Duration of response (months)			
Median (95% CI)	28.4 (21.6, 42.9)	23.5 (16.8, 35.0)	42.9 (23.7, 63.0)
Events [*n*, (%)]	58 (59.8)	40 (63.5)	18 (52.9)
Progression‐free survival (months)			
Median (95% CI)	22.9 (19.3, 28.1)	19.3 (14.2, 25.0)	31.5 (22.0, 48.0)
Events [*n*, (%)]	105 (64.0)	76 (65.5)	29 (60.4)
Overall survival (months)			
Median (95% CI)	58.1 (45.7, 71.7)	50.0 (33.7, 68.8)	83.7 (45.7, NA)
Events [*n*, (%)]	85 (51.8)	62 (53.4)	23 (47.9)
Time to second‐line anti‐myeloma therapy (months)			
Median (95% CI)	29.7 (23.2, 37.5)	26.6 (19.3, 37.5)	30.6 (23.0, 46.0)
Events [*n*, (%)]	94 (57.3)	63 (54.3)	31 (64.6%)

*Note:* 24‐month progression‐free survival rate (24‐month PFS‐rate) was defined as the rate of patients who were alive and free of disease progression (according to investigator's assessment) at 24 months after start of treatment with lenalidomide. The 24‐month PFS‐rate was estimated using the Kaplan–Meier method. Patients without disease progression before the onset of a subsequent anti‐myeloma treatment were censored at the start date of the subsequent treatment. Patients alive without disease progression and without any subsequent anti‐myeloma treatment at the end of observation period were censored at the date of last contact.

Abbreviations: CI, confidence interval; FAS, full analysis set; PFS, progression‐free survival; Rd., lenalidomide/dexamethasone.

^a^ Overall response rate was defined as the proportion of patients with documented sCR, CR, VGPR or PR as best response under Rd therapy (treatment observation period) in relation to all evaluable patients.

^b^ Best response was documented during the 24‐months treatment observation period.

### 3.4 Effectiveness in Pre‐Defined Subgroups

In the subgroup of patients > 75 years, the 24‐month PFS‐rate was 40.1% (95% CI 29.9, 50.1) vs. 66.3% (95% CI 49.6, 78.5) in patients ≤ 75 years. ORRs were 54.3% (95% CI 45.3, 63.1) and 70.8% (95% CI 56.7, 81.8), respectively. The median DOR was shorter in the subgroup of patients > 75 years [23.5 months (95% CI 16.8, 35.0) vs. 42.9 months (95% CI 23.7, 63.0)] (Table 2). Median PFS and OS were 19.3 months (95% CI 14.2, 25.0) and 50.0 months (95% CI 33.7, 68.8) in patients > 75 years, and 31.5 months (95% CI 22.0, 48.0) and 83.7 months (95% CI 45.7, NA) in patients ≤ 75 years (Table 2, Figure 2).

In the subgroup of impaired patients (G8‐GA score ≤ 14), ORR was 53.5% (95% CI 43.0, 63.7) vs. 66.7% (95% CI 49.5, 80.3) in non‐impaired patients [G8‐GA missing: 64.4% (95% CI 49.8, 76.8)]. Median PFS and OS were 19.3 months (95% CI 13.7, 25.2) and 44.1 months (95% CI 27.2, 60.1) in impaired patients, and 45.6 months (95% CI 19.3, 66.5) and 84.6 months (95% CI 52.5, NA) in non‐impaired patients (Figure 2).

The median PFS was shorter in the subgroup of patients with severe [4.4 months (95% CI 1.6, 23.0)] or moderate RI [19.4 months (95% CI 15.5, 25.4)] compared to that of patients with mild [28.1 months (95% CI 21.6, 50.0)] or no RI [48.0 months (10.8, NA)]. The corresponding median OS was 8.8 months (95% CI 1.6, NA), 46.2 months (95% CI 30.3, 68.8), 84.2 months (95% CI 52.5, NA), and not reached (95% CI 47.7, NA), respectively (Figure 2, Table S5).

### 3.5 Impact of Covariates as Predictors for PFS

Multivariate Cox regression analysis with pre‐defined covariates as potential predictors for PFS (*n* = 57/159 censored cases) suggested statistically significant improved outcome in patients with better renal function (per increase of CrCl value by 10 mL/min steps: HR = 0.85; 95% CI 0.77–0.93; *p* < 0.001), and higher pre‐treatment level of hemoglobin (Hb) (per increase of Hb level by 1 g/dL steps: HR = 0.89; 95% CI 0.8–0.98; *p* = 0.016). A higher ECOG performance status at baseline (≥ 2 vs. 0/1) was observed as a negative predictor of PFS (HR = 2.72; 95% CI: 1.64–4.54; *p* < 0.001) (Table S6). Corresponding effectiveness data (PFS/OS) are shown in the Table S7.

### 3.6 Quality of Life

**FIGURE 3:**
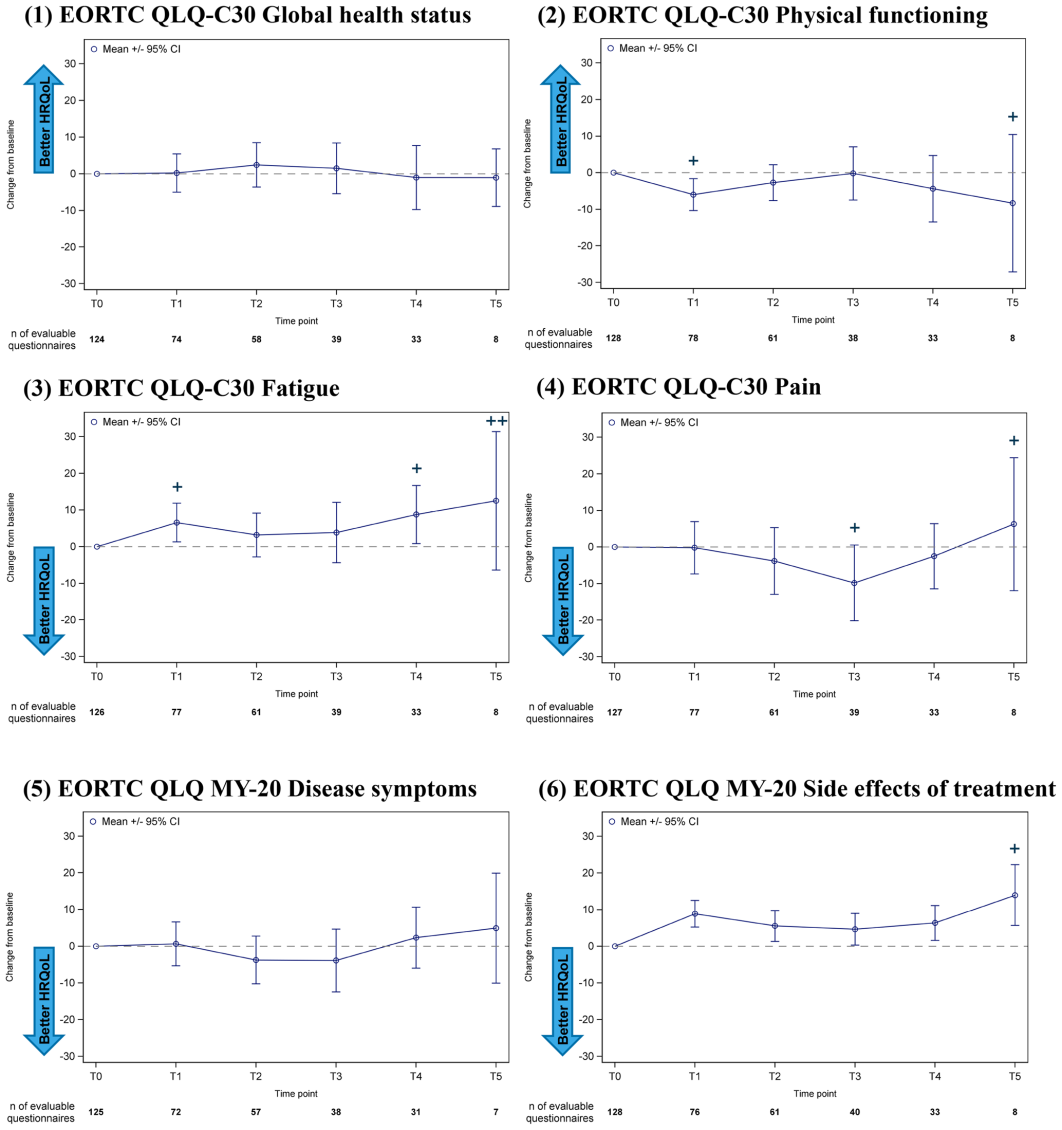
Longitudinal analysis of mean (StD) HRQoL score changes from baseline over time (24‐month treatment observation period). (1)–(4) EORTC QLQ‐C30. (5)–(6) EORTC QLQ‐MY20. Circles: Mean; whisker: 95% CI. T0: Baseline; T1: Month 3; T2: Month 6; T3: Month 12; T4: Month 18; T5: Month 24 (after start of Rd treatment). EORTC QLQ‐C30 and QLQ‐MY20 total score range from 1 to 100. Minimal important difference (MID) thresholds according to the established definition of MID for EORTC QLQ‐C30 and QLQ‐MY20: global health status (small deterioration −5 to −10); physical functioning (small deterioration −5 to −10); pain (small deterioration −3 to −11; small improvement +5 to +9; medium improvement +9 to +14); fatigue (small deterioration −5 to −10; medium deterioration −10 to −15); disease symptoms (deterioration −10); and side effects of treatment (deterioration −10). “+” and “++” indicate mean value outside of the proposed range of MID (“small” and “medium deterioration”, respectively). CI, confidence interval; EORTC, European Organization for Research and Treatment of Cancer; FAS, full analysis set; HRQoL, health‐related quality‐of‐life.

At baseline, the questionnaire return rate was 86.6%. The EORTC QLQ‐C30 GHS scores remained stable during the observation period indicating that there were no clinically relevant changes in HRQoL. Moreover, EORTC QLQ‐C30 and QLQ‐MY20 subscale scores showed only minor changes (Figure 3). Details on questionnaire return rates and changes of scores from baseline are available in supporting appendix (Tables S8 and S9).

### 3.7 Safety

Adverse events were analyzed in the safety analysis population (SAF) and are summarized in Table 4. Overall, 165 (98.2%) patients were reported with at least one treatment‐emergent adverse event (AE). Grade 3/4 AEs were documented in 108 (64.3%) patients, 96 (57.1%) patients experienced at least one serious adverse event (SAE). A total of 85 (50.6%) patients deceased during the study, of which 15 (8.9%) patients died during the TOP. The most common grade 3/4 AEs of specific interest were hematologic events (16.7%), infections (16.1%), and cardiac events (11.3%). Pneumonia was the only SAE reported in > 5% of patients (*n* = 11; 6.5%). An overview of documented AE of all grades (≥ 5%) and AE of specific interest is provided in the supporting appendix (Tables S10 and S11, respectively).

**TABLE 4:** Treatment‐emergent adverse events (SAF).

Adverse event	Total population (*N* = 168)	> 75 years (*N* = 116)	≤ 75 years (*N* = 52)
Any adverse event [*n*, (%)]			
Any event	165 (98.2)	115 (99.1)	50 (96.2)
Grade 3/4	108 (64.3)	72 (62.1)	36 (69.2)
SAE	96 (57.1)	67 (57.8)	29 (55.8)
Pneumonia	11 (6.5)	7 (6.0)	4 (7.7)
SAE with fatal outcome	15 (8.9)	10 (8.6)	5 (9.6)
Hematologic events^a^ [*n*, (%)]			
Any event	78 (46.4)	56 (48.3)	22 (42.3)
Grade 3/4	28 (16.7)	18 (15.5)	10 (19.2)
SAE	8 (4.8)	4 (3.4)	4 (7.7)
Infections [*n*, (%)]			
Any event	68 (40.5)	42 (36.2)	26 (50.0)
Grade 3/4	27 (16.1)	17 (14.7)	10 (19.2)
Pneumonia	8 (4.8)	5 (4.3)	3 (5.8)
SAE	30 (17.9)	19 (16.4)	11 (21.2)
Cardiac events^b^ [*n*, (%)]			
Any event	29 (17.3)	16 (13.8)	13 (25.0)
Grade 3/4	19 (11.3)	10 (8.6)	9 (17.3)
SAE	21 (12.5)	12 (10.3)	9 (17.3)
Lenalidomide‐related AE^c^ [*n*, (%)]			
Any grade	123 (73.2)	87 (75.0)	36 (69.2)
Grade 3/4 (any event)	45 (26.8)	27 (23.3)	18 (34.6)
Thrombocytopenia	9 (5.4)	6 (5.2)	3 (5.8)
Anemia	7 (4.2)	5 (4.3)	2 (3.8)
SAE [*n*, (%)]			
Any event	23 (13.7)	15 (12.9)	8 (15.4)
Anemia	3 (1.8)	2 (1.7)	1 (1.9)
Acute kidney injury	2 (1.2)	2 (1.7)	0 (0.0)
Deep vein thrombosis	2 (1.2)	1 (0.9)	1 (1.9)
Pulmonary embolism	2 (1.2)	2 (1.7)	0 (0.0)
SAE with fatal outcome [*n*, (%)]			
Acute kidney injury	1 (0.6)	1 (0.9)	0 (0.0)
Dexamethasone‐related AE^d^ [*n*, (%)]			
Any grade	48 (28.6)	33 (28.4)	15 (28.8)
Grade 3/4	10 (6.0)	7 (6.0)	3 (5.8)
SAE	6 (3.6)	4 (3.4)	2 (3.8)
Treatment modification due to AE [*n*, (%)]			
Lenalidomide			
Dose reduction	44 (26.2)	25 (21.6)	19 (36.5)
Interruption	96 (57.1)	65 (56.0)	31 (59.6)
Discontinuation	70 (41.7)	48 (41.4)	22 (42.3)
Due to toxicity	50 (29.8)	35 (30.2)	15 (28.8)
Dexamethasone			
Dose reduction	20 (11.9)	15 (12.9)	5 (9.6)
Interruption	80 (47.6)	58 (50.0)	22 (42.3)
Discontinuation	63 (37.5)	42 (36.2)	21 (40.4)
Due to toxicity	12 (7.1)	7 (6.0)	5 (9.6)

*Note:* The table is sorted by number of patients with the corresponding AE in the analysis set. A treatment‐related AE (adverse drug reaction) was defined as an AE related to lenalidomide or dexamethasone as assessed by the treating physician. MedDRA v26.0 was used for classification of the reported AE.

Abbreviations: AE, adverse event; CTCAE, common terminology criteria for adverse events; MedDRA, medical dictionary for regulatory activities; PT, preferred term; SAE, serious adverse event; SAF, safety analysis set.

^a^ Hematologic adverse events were defined as SOC “blood and lymphatic system disorders” and PTs “hemoglobin decreased”, “neutrophil count decreased”, “platelet count decreased”, “white blood cell count decreased”, and “bone marrow band”.

^b^ Cardiac disorders were defined as SOC “cardiac disorders”, HLT “cardiac valve therapeutic procedures”, and PTs “congenital aortic valve stenosis”, and “coronary arterial stent insertion”.

^c^ Lenalidomide‐related grade 3/4 AEs occurring in at least 3% of the total population (SAF) are displayed, except for SAE (occurring in at least 1% of the SAF) and AE with fatal outcome (all reported cases are shown).

^d^ None of the documented dexamethasone‐related grade 3/4 AEs (PT) were observed in more than 2 patients (0.6%); the incidence of all reported dexamethasone‐related SAEs (PT) was < 1%; dexamethasone‐related SAEs with fatal outcomes were not reported.

Grade 3/4 AEs related to lenalidomide treatment were reported for 45 (26.8%) patients; the most common grade 3/4 AEs were thrombocytopenia (5.4%) and anemia (4.2%) (Table 4). Lenalidomide‐related SAE were reported for 23 (13.7%) patients, with anemia (*n* = 3; 1.8%) being the most frequently reported event. One patient (0.6%) was reported by the treating physician with a fatal lenalidomide‐related SAE (acute kidney injury).

Overall, 10 (6.0%) patients were documented with grade 3/4 AEs related to dexamethasone treatment, while dexamethasone‐related SAEs were rarely observed (*n* = 6; 3.6%).

Patients with grade 3/4 lenalidomide‐related AEs were reported less frequently in the subgroup of patients > 75 years (*n* = 27; 23.3%) compared to the patients ≤ 75 years (*n* = 18; 34.6%), while lenalidomide‐related SAEs were reported at similar frequencies in both subgroups (Table 4). Based on the G8‐GA subgroup analyses, lower incidences of grade 3/4 lenalidomide‐related AEs and SAEs were observed in the subgroup of non‐impaired patients [(*n* = 8; 23.5% and *n* = 3; 8.8%)] compared to the impaired patients [(*n* = 25; 28.1% and *n* = 12; 13.5%)]. A comparison of the subgroup analyses stratified by age and G8‐GA is presented in the supporting appendix (Table S13).

Lenalidomide and dexamethasone dose reductions due to AEs were documented with 44 (26.2%) and 20 (11.9%) patients, respectively. Treatment interruptions were also common (*n* = 96; 57.1% and *n* = 80; 47.6%, respectively) (Table 4). Permanent discontinuation of lenalidomide treatment due to toxicity was reported for 50 (29.8%) patients at similar frequencies in the subgroups stratified by age (> 75 vs. ≤ 75 years: *n* = 35; 30.2% vs. *n* = 15; 28.8%), while discontinuation of dexamethasone due toxicity was reported less common (*n* = 12; 7.1%).

In total, 15 cases of second primary malignancies (SPM) were reported in 12 (7.1%) patients [hematologic tumor: *n* = 2 (1.2%), solid tumor: *n* = 13 (7.7%); refer to the supporting appendix (Table S12)].

## 4 Discussion

Our real‐world data support lenalidomide and low‐dose dexamethasone (Rd) as effective and safe frontline treatment in patients with transplant‐ineligible, newly diagnosed multiple myeloma (TIE NDMM) of all ages, as previously reported for the pivotal phase III FIRST trial [1, 2, 10, 12].

The 24‐month PFS‐rate was 48%, while it was not published for the FIRST trial. The median TTR observed in our real‐world population was longer than that reported for the pivotal trial (3.1 vs. 1.8 months) [1], most probably reflecting clinical routine practice [36]. Consistent with the shorter duration of lenalidomide treatment observed in our NIS, the ORR was apparently lower (59%) compared to the pivotal trial (81% and 79% for continuous and fixed‐duration lenalidomide treatment, respectively) [2]. However, the median DOR, time to second‐line anti‐myeloma therapy, and, most notably, the median PFS and OS were within a similar range to those reported in the pivotal trial [1, 2]. The proportion of patients in our NIS receiving second‐line anti‐myeloma therapy (most commonly bortezomib or lenalidomide‐based treatment regimens) was also similar to the pivotal trial, which may have contributed to the observed survival outcomes [2].

In contrast to the FIRST trial [1], our real‐world study included a substantially higher proportion of patients older than 75 years (71% vs. 35%), while fewer patients presented with ISS stage III at baseline (24% vs. 40%). Given the heterogeneity of older patients [3] and the poorer prognosis typically associated with ISS stage III, it remains uncertain to what extent these differences may have influenced our results. Apart from these differences, other patient and disease characteristics in our study population were similar to those observed in the pivotal trial.

When stratifying outcomes by age (> 75 years vs. ≤ 75 years), we observed similar long‐term outcomes (median PFS and OS) to those reported in the pivotal trial [2, 11]. Interestingly, both studies achieved very similar median PFS and OS in patients older than 75 years, despite a markedly shorter median treatment duration in our real‐world approach. This observation may suggest that a comparatively short duration of lenalidomide treatment may be associated with similar long‐term outcomes as seen in the pivotal trial, possibly at the expense of a lower ORR.

Our results further supported Rd as a viable treatment option for patients with mild to moderate renal impairment in clinical routine [13], as the median PFS observed in these subgroups was similar to that reported in the pivotal trial [12]. In addition, a multivariate Cox regression analysis indicated that better renal function and higher pre‐treatment hemoglobin levels were associated with a lower risk of PD or death. Conversely, a higher ECOG performance status score (≥ 2 vs. 0/1) at baseline was observed as a negative predictor of PFS. It is important to emphasize that these associations do not establish causality, given the observational nature of our study. Nonetheless, our findings are consistent with results from other (retrospective) real‐world studies investigating Rd in patients with TIE NDMM [37, 38, 39, 40].

Nowadays, in the era of monoclonal antibody therapies, the combination of daratumumab with Rd (DaraRd) is considered a standard of care across all ages groups [16], as the triplet‐regimen demonstrated superior PFS and OS compared to Rd alone [17, 21, 22, 41]. Moreover, subgroup analysis of the MAIA study showed a significant benefit in PFS for frail patients (median: 52.2 months vs. 30.4 months), with a median age of 77 years [18]. However, the rates of deaths among frail patients were similar [42], and so far it remains unclear whether the addition of daratumumab will lead to superior survival as well. This is particularly important given that real‐world data show that up to 57% of patients diagnosed with TIE NDMM only receive one line of therapy [43]. Therefore, the choice of the most effective treatment regimen upfront is of major importance, especially for very elderly and frail patients. Given the potential benefits of the DaraRd triplet regimen in terms of PFS in elderly frail patients [18, 42], the oral and self‐administering outpatient Rd treatment may be still appropriate for a cautious disease control approach that emphasizes quality‐of‐life rather than targeting complete response and OS.

To further assess patients' impairment status at baseline, we utilized the validated G8 Geriatric Assessment (G8‐GA) tool in our study, as formerly recommended by the European Organization for Research and Treatment of Cancer (EORTC) Cancer in the Elderly Task Force (ETF) [7, 24, 25]. Our findings using the G8‐GA tool were similar to those of the FIRST trial, although it is important to note that different geriatric assessment tools were used across the studies [10]. In our study, patients classified as impaired by G8‐GA (score ≤ 14) had substantially shorter median PFS (19.3 months) and OS (44.1 months) compared to the non‐impaired patients (45.6 and 84.6 months, respectively). This difference aligns with the observation that impaired patients had worse baseline prognosis as indicated by key patient and disease characteristics. Notably, the median age was similar between impaired and non‐impaired subgroups (78.8 years vs. 76.4 years), suggesting that the G8‐GA tool may capture aspects of vulnerability not reflected by age alone. Furthermore, non‐impaired patients (G8‐GA score > 14) experienced fewer adverse events compared to impaired patients, and their rates of adverse events were similar to those observed in younger patients (≤ 75 years) in our study. The prognostic value of the G8‐GA was further supported by our observation that non‐impaired patients, despite being approximately 5 years older on average than the subgroup aged ≤ 75 years, had longer median PFS (45.6 vs. 31.5 months) and similar overall survival (84.6 vs. 83.7 months).

Thus, our results suggest that the G8‐GA may be a useful tool for predicting clinical long‐term outcomes in elderly patients with TIE NDMM in routine clinical practice. However, given the observational nature of our study, further prospective research is warranted to confirm the predictive value of the G8‐GA in this setting.

No clinically meaningful changes in QoL and Global Health Status (GHS) were observed in the NIS, which is in line with the GHS results reported in the pivotal trial [15]. However, our data on patient‐reported HRQoL indicate that, in clinical routine, disease symptoms and side effects were generally manageable by the treating physicians, even in a predominantly older patient population compared to that of the pivotal trial.

In our study, Rd treatment was well tolerated, including patients older than 75 years and those identified as impaired by the G8 Geriatric Assessment. As anticipated for a predominantly elderly patient population, most patients over 75 years of age (86%) began dexamethasone treatment at a reduced starting dose of 20 mg or less, whereas only 44% of patients aged 75 years or younger received the recommended starting dose of 40 mg. Additionally, a substantial proportion of patients (45%) started lenalidomide treatment at a reduced dosage, including 35% of those with mild or no renal impairment (CrCl ≥ 60 mL/min). Thus, our results were similar to the findings of other real‐world studies investigating Rd as frontline treatment in patients with TIE NDMM [37, 38, 44] and suggest that individualized, cautious Rd treatment management may achieve effective lenalidomide dosing in clinical practice similar to that reported in the pivotal FIRST trial [10, 12]. While dose modifications and reduced dose intensity were common in our real‐world study, it is worth mentioning that we did not perform a formal correlation analysis between dose intensity and clinical outcomes such as ORR or PFS. Future studies are warranted to further elucidate the impact of dose intensity on treatment effectiveness in this patient population.

Hematologic events, infections, and cardiac events were among the most commonly reported adverse events (AE) in our study. The prevalence of grade 3/4 AE (64.3%) was lower than reported in the pivotal trial [1], but similar to a (retrospective) real‐world investigation with TIE NDMM (65.1%) [38]. The incidence of grade 3/4 hematological events (17%) in our NIS was lower than in the FIRST trial [1, 2], however, with only limited and variated real‐world data available [38, 44].

High‐grade infections, particularly those with a fatal outcome, are a major concern of Rd treatment [45]. In our study, 16% of patients experienced grade 3/4 infections, a rate that is consistent with findings (14.3%) from a large meta‐analysis of phase II/III clinical trials [46] and a real‐world investigation (17%) reported by Del Fabro et al. [38].

Notably, results from the randomized phase III RV‐MM‐PI‐0752 trial [47], which included predominantly elderly (median age: 77 years) and intermediate‐frail patients who are commonly excluded from clinical trials, also showed similar rates of grade 3/4 hematologic events (20%) and infections (12%) in the Rd control arm. In that trial, grade 3/4 pneumonia was the most frequently reported severe infection (4.0%), which aligns with the rate observed in our study (4.8%) and another real‐world analysis reported by Del Fabro et al. (3.4%) [38, 47]. Furthermore, the rate of grade 3/4 cardiac events (11.3%) observed in our real‐world population was within the expected range for a predominantly elderly and frail population and similar to those observed in the pivotal trial [1, 10].

In the FIRST‐NIS, 57% of patients experienced at least one serious adverse event (SAE), with pneumonia being the most commonly documented event. This result is in line with the results observed in the Rd control arms in large phase III clinical trials [17, 48]. The frequency of AE that resulted in death was also similar to those reported in phase III clinical trials [17, 42, 47, 48]. The prevalence of grade 3/4 lenalidomide‐related AE (26.8%) was lower than reported in the pivotal trial [1], which may be attributable to the shorter duration of lenalidomide treatment observed in our real‐world population. Previous studies have described an increase in the incidence of severe adverse events with longer treatment duration [1], which may, at least in part, explain the moderately increased rate of adverse events among patients aged 75 years or younger in our study.

SAE related to lenalidomide were documented in 23 (13.7%) patients, including one fatal case (0.6%) of acute kidney injury. Discontinuation from lenalidomide treatment due to toxicity was observed in approximately one third of the patients, regardless of age.

Thus, both the frequency and severity of AEs, including second primary malignancies, reflected the known lenalidomide safety profile [23] and importantly, no new safety signals emerged.

## 5 Limitations

When interpreting the results of our NIS, it is important to note certain limitations. The non‐interventional setting of FIRST‐NIS limits the direct comparison of the effectiveness and safety data to those published for the pivotal trial due to the different study settings and heterogeneity of the study populations; all cross‐trial comparisons are of a purely descriptive nature and must be interpreted with caution [49]. The number of available HRQoL questionnaires decreased across all timepoints, which limits the interpretability of the data at later time points. Pre‐defined (non‐randomized) subgroup analyses were of exploratory nature, and small group sizes may limit interpretability in certain clinically relevant subgroups.

Despite these limitations, the results of our prospective FIRST‐NIS complements the evidence from the pivotal FIRST trial and real‐world investigations by providing insights into the effectiveness of frontline Rd in an unselected predominantly elderly and frail population of patients with TIE NDMM and thus may contribute to treatment decision‐making in routine clinical practice, particularly in patients who are not suitable for multi‐drug regimens according to the current standard of care [16]. Furthermore, the comprehensive and prospective study design and the long‐term follow‐up render the FIRST‐NIS a valuable addition to the results published from the pivotal trial.

## 6 Conclusion

In summary, the results of the FIRST‐NIS support Rd as an effective and safe frontline treatment option for patients with TIE NDMM, irrespective of age, with similar clinical outcomes in the real world compared to the pivotal trial. Interestingly, our results suggest that the G8‐GA tool may be suitable to identify impaired and non‐impaired patients and to predict clinical long‐term outcome in patients with TIE NDMM. A higher pre‐treatment hemoglobin level was associated with better survival. Rd may still be a viable treatment option even in patients with RI. Patient‐reported HRQoL was maintained throughout Rd treatment. No new safety signals emerged. Thus, in clinical practice, Rd may represent an attractive alternative as frontline treatment for patients with TIE NDMM and ineligible for triplet or quadruplet regimens.

## Author Contributions

**H. Nückel:** conceptualization, methodology, investigation, resources, writing – review and editing, supervision. **T. Behlendorf:** investigation, resources, writing – review and editing. **H. Schulz:** investigation, writing – review and editing, resources. **M. Schulze:** writing – review and editing, investigation, resources. **C. Schardt:** investigation, writing – review and editing, resources. **T. Medinger:** conceptualization, writing – review and editing, methodology, formal analysis, visualization. **M. Koenigsmann:** investigation, writing – review and editing, resources. **T. Dechow:** investigation, writing – review and editing, resources. **M. Indorf:** writing – original draft, project administration, methodology, visualization. **D. Bürkle:** investigation, writing – review and editing, resources. **V. Engelbertz:** investigation, writing – review and editing, resources. **J. Rauh:** investigation, writing – review and editing, resources. **B. Schmidt:** investigation, writing – review and editing, resources. **A. Sauer:** investigation, writing – review and editing, resources. **C. Vannier:** conceptualization, methodology, writing – review and editing. **K. Potthoff:** conceptualization, methodology, writing – review and editing, supervision.

## Funding

This study was financially supported by Celgene GmbH/BMS GmbH & Co. KGaA. None of the funders had any role in study design, data collection and analysis, interpretation of results, decision to publish or preparation of the manuscript.

## Ethics Statement

The FIRST‐NIS was approved by the responsible ethics committee (*Ethik‐Kommission bei der Landesärztekammer Baden‐Württemberg*, project number F‐2015‐030). All experiments comply with the current laws in Germany, where they were performed. All procedures performed in studies involving human participants were in accordance with the ethical standards of the national research committee and with the 1964 Helsinki declaration and its later amendments.

## Consent

Written informed consent was obtained from all patients.

## Conflicts of Interest

H. Schulz, M. Schulze, C. Schardt, T. Medinger, M. Koenigsmann, T. Dechow, M. Indorf, J. Rauh, A. Sauer, C. Vannier, K. Potthoff: No conflict of interest. H. Nückel: Honoraria: Sanofi‐Aventis; Johnson‐Johnson. T. Behlendorf: Honoraria (Lectures and educational sessions): Sachsen‐anhaltische Krebsgesellschaft, Esteve; Support for meetings/events: Ipsen, Beigene; Stock or stock options: BMS, Tilray, Amgen, Incyte, J&J, Biontech. D. Bürkle: Support for meetings/events: Merck; Advisory Boards: Lilly, Merck. V. Engelbertz: Honoraria: Roche, BMS, Janssen, Lilly; Support for meetings/events: Astra Zeneca, AOP, Roche, Janssen, BMS; Advisory Boards: Beigene, Amgen, AstraZeneca, BMS, Kite/Gilead, Lilly, BI. B. Schmidt: Honoraria (Consulting fees): AbbVie, AOP Orphan, AstraZeneca, BeiGene, BMS, Hexal, Incyte, Janssen‐Cilag, Novartis, Oncopeptides, Pfizer, Sanofi, and SoBi; Lectures/manuscript writing: AstraZeneca; Case presentations: AbbVie, AstraZeneca, SoBi; Support for meetings/events: AbbVie, BeiGene, AstraZeneca.

## Supporting information


**Table S1:** FIRST‐NIS versus FIRST trial—Patient demographic and clinical characteristics, treatment characteristics, and clinical outcome (total population). Data displayed with descriptive statistics [median (range) or frequencies (%)] for the FIRST‐NIS (FAS; *n* = 164) and ITT populations of the Rd continuous (Rdc) and Rd18 treatment arms (FIRST‐trial).
**Table S2:** FIRST‐NIS versus FIRST trial—Patient demographic and clinical characteristics, treatment characteristics, and clinical outcome in patients aged older than 75 years. Data displayed with descriptive statistics [median (range) or frequencies (%)] for the FIRST‐NIS (FAS; *n* = 164) and ITT populations of the Rd continuous (Rdc) and Rd18 treatment arms (FIRST‐trial).
**Table S3:** FIRST‐NIS versus FIRST trial—Patient demographic, clinical and treatment characteristics stratified by G8‐geriatric assessment (impairment status) and simplified frailty index (frailty status). Data displayed with descriptive statistics [median (range) or frequencies (%)] for the FIRST‐NIS (FAS; *n* = 164) and ITT populations of the pooled Rd continuous (Rdc) and Rd18 treatment arms stratified by frailty status (FIRST‐trial).
**Table S4:** FIRST‐NIS versus FIRST trial—Clinical outcome stratified by G8‐Geriatric assessment (impairment status) and simplified frailty index (frailty status). Data displayed with descriptive statistics [median (range) or frequencies (%)] for the FIRST‐NIS (FAS; *n* = 164) and ITT populations of the Rd continuous (Rdc) and Rd18 treatment arms stratified by frailty status (FIRST‐Trial).
**Table S5:** Renal impairment—selected patient and treatment characteristics stratified by creatinine clearance. Data stratified by CrCl displayed with descriptive statistics [median (range) or frequencies (%)] for the SAF (*n* = 168).
**Table S6:** Multivariate Cox regression analysis. Multivariate Cox regression analysis with pre‐defined covariates as potential predictors for PFS.
**Table S7:** Progression‐free survival and overall survival stratified by ECOG PS, CrCl, and hemoglobin at baseline. Data displayed with descriptive statistics [median (range) or frequencies (%)] stratified by ECOG PS, CrCl, and hemoglobin at baseline (FAS).
**Table S8:** Return and evaluability of questionnaires. Return of questionnaires.
**Table S9:** Changes from baseline in patient‐reported quality of life (QoL) questionnaire score. Changes from baseline in six clinically relevant patient‐reported quality of life (QoL) questionnaire scores. Data displayed with numbers of observations, mean and standard deviation (StD). Higher scores in the global health status/QoL and functional scales (physical function) correspond to higher perceived QoL/healthy level of functioning, while higher scores in the symptom scales (fatigue, pain, disease symptoms, side effects of treatment) correspond to higher level of perceived symptoms severity based on the questionnaire results.
**Table S10:** Treatment‐emergent adverse events (AEs) occurring in > 5% (any grade) of the safety population (SAF: *N* = 168). Data displayed with descriptive statistics [frequencies (%)] for the SAF (*n* = 168).
**Table S11:** Selected treatment‐emergent adverse events (AEs) in ≥ 1% of patients with either grade 3/4 events or ≥ 1 patient with grade 5 event (SAF: *N* = 168). Data displayed with descriptive statistics [frequencies (%)] for the SAF (*n* = 168).
**Table S12:** Second primary malignancies during or after lenalidomide treatment (SAF). Data displayed with descriptive statistics [frequencies (%)] for the SAF (*n* = 168).
**Table S13:** Comparison of subgroup analyses stratified by G8‐Geriatric assessment vs. age (> 75 vs. ≤ 75 years) [FAS/SAF]. Data displayed with descriptive statistics [median (range) or frequencies (%)] (SAF; *n* = 168).

## Data Availability

The data supporting the findings of the study are not openly available due to data privacy protection regulations. Enquiries regarding the data used for this study can be directed to the corresponding author.
