# Supplementary material for: Lenalidomide Plus Dexamethasone as FIRST‐Line Therapy in Transplant‐Ineligible Patients With Multiple Myeloma: Final Results of the Prospective, Non‐Interventional Study FIRST‐NIS and Comparison With the FIRST Pivotal Phase III Clinical Trial
Source: Cancer Med. 2026 Apr 2;15(4):e71758. doi: 10.1002/cam4.71758 (PMC13052119; doi:10.1002/cam4.71758)
Supplement: Supplementary file 1 — Table S1: FIRST‐NIS versus FIRST trial—Patient demographic and clinical characteristics, treatment characteristics, and clinical outcome (total population). Data displayed with descriptive statistics [median (range) or frequencies (%)] for the FIRST‐NIS (FAS; n = 164) and ITT populations of the Rd continuous (Rdc) and Rd18 treatment arms (FIRST‐trial). Table S2: FIRST‐NIS versus FIRST trial—Patient demographic and clinical characteristics, treatment characteristics, and clinical outcome in patients aged older than 75 years. Data displayed with descriptive statistics [median (range) or frequencies (%)] for the FIRST‐NIS (FAS; n = 164) and ITT populations of the Rd continuous (Rdc) and Rd18 treatment arms (FIRST‐trial). Table S3: FIRST‐NIS versus FIRST trial—Patient demographic, clinical and treatment characteristics stratified by G8‐geriatric assessment (impairment status) and simplified frailty index (frailty status). Data displayed with descriptive statistics [median (range) or frequencies (%)] for the FIRST‐NIS (FAS; n = 164) and ITT populations of the pooled Rd continuous (Rdc) and Rd18 treatment arms stratified by frailty status (FIRST‐trial). Table S4: FIRST‐NIS versus FIRST trial—Clinical outcome stratified by G8‐Geriatric assessment (impairment status) and simplified frailty index (frailty status). Data displayed with descriptive statistics [median (range) or frequencies (%)] for the FIRST‐NIS (FAS; n = 164) and ITT populations of the Rd continuous (Rdc) and Rd18 treatment arms stratified by frailty status (FIRST‐Trial). Table S5: Renal impairment—selected patient and treatment characteristics stratified by creatinine clearance. Data stratified by CrCl displayed with descriptive statistics [median (range) or frequencies (%)] for the SAF (n = 168). Table S6: Multivariate Cox regression analysis. Multivariate Cox regression analysis with pre‐defined covariates as potential predictors for PFS. Table S7: Progression‐free survival and overall survival stratified b [file CAM4-15-e71758-s001.docx]

# FIRST-NIS Supplementary data

This appendix has been provided by the authors to provide additional information about their work.

Supplement to:

Nückel H, Behlendorf T, Schulz H, Schulze M, Schardt C, Medinger T, Koenigsmann M, Dechow T, Indorf M, Bürkle D, Engelbertz V, Rauh J, Schmidt B, Sauer A, Vannier C, Potthoff K

Lenalidomide plus dexamethasone as first line therapy in transplant-ineligible patients with multiple myeloma: Final results of the prospective, non-interventional study FIRST-NIS and comparison with the FIRST pivotal phase III clinical trial.

Cancer Med. 2025; XXXX, DOI: XXXX

**Table of Contents**

[**Table of Contents** 2](#_Toc191894611)

[**1.** **FIRST-NIS vs. FIRST Trial** 3](#_Toc191894612)

[1.1 Patient characteristics, lenalidomide-treatment, and clinical outcome (total population) 3](#_Toc191894613)

[1.2 Patient characteristics, lenalidomide-treatment and clinical outcome in patients aged >75 years 5](#_Toc191894614)

[1.3 G8-Geriatric assessment tool vs. Simplified frailty Index 6](#_Toc191894615)

[**2.** **Methods** 8](#_Toc191894616)

[2.1 Definition of secondary effectiveness endpoints 8](#_Toc191894617)

[**3.** **Renal impairment – Patient and treatment characteristics** 9](#_Toc191894619)

[3.1 Renal impairment - Selected patient and treatment characteristics stratified by CrCl (mL/min) 9](#_Toc191894620)

[**4.** **Multivariate COX regression analysis** 11](#_Toc191894621)

[4.1 Covariates as predictors for PFS 11](#_Toc191894622)

[4.2 PFS and OS stratified by ECOG PS, creatinine clearance, and hemoglobin at baseline 12](#_Toc191894623)

[**5.** **Health-related Quality of Life (QoL)** 13](#_Toc191894624)

[5.1 Return and evaluability of patient-reported QoL questionnaires 13](#_Toc191894625)

[5.2 Changes from baseline in patient-reported quality of life (QoL) questionnaire score 14](#_Toc191894626)

[**6. Adverse events 15**](#_Toc191894627)

[6.1 Adverse events (all grades) 15](#_Toc191894628)

[6.2 Adverse events of specific interest 16](#_Toc191894629)

[6.2 Second primary malignancies 17](#_Toc191894630)

[**7.** **Subgroup analyses - G8-Geriatric assessment vs. age (>75 vs. ≤75 years)** 18](#_Toc191894631)

[**8.** **References** 20](#_Toc191894632)

**List of Supplementary Tables**

**[Supplementary Table S1](#_Toc191894733)** [FIRST-NIS vs. FIRST Trial -](#_Toc191894733)[Patient demographic and clinical characteristics, treatment characteristics, and clinical outcome (total population) 3](#_Toc191894733)

[**Supplementary Table S2** FIRST-NIS vs. FIRST Trial - Patient demographic and clinical characteristics, treatment characteristics, and clinical outcome in patients aged older than 75 years 5](#_Toc191894734)

[**Supplementary Table S3** FIRST-NIS vs. FIRST Trial - Patient demographic, clinical and treatment characteristics stratified by G8-geriatric assessment (impairment status) and simplified frailty index (frailty status) 6](#_Toc191894735)

[**Supplementary Table S4** FIRST-NIS vs. FIRST Trial - Clinical outcome stratified by G8-Geriatric assessment (impairment status) and simplified frailty index (frailty status) 7](#_Toc191894736)

[**Supplementary Table S5** Renal impairment – selected patient and treatment characteristics stratified by creatinine clearance 9](#_Toc191894737)

[**Supplementary Table S6** Multivariate Cox regression analysis 11](#_Toc191894738)

[**Supplementary Table S7** Progression-free survival and overall survival stratified by ECOG PS, CrCl, and hemoglobin at baseline 12](#_Toc191894739)

[**Supplementary Table S8** Return and evaluability of questionnaires 13](#_Toc191894740)

[**Supplementary Table S9** Changes from baseline in patient-reported quality of life (QoL) questionnaire score 14](#_Toc191894741)

[**Supplementary Table S10** Treatment-emergent adverse events (AEs) occurring in >5% (any grade) of the safety population (SAF: N=168) 15](#_Toc191894742)

[**Supplementary Table S11** Selected treatment-emergent adverse events (AEs) in ≥1% of patients with either grade 3/4 events or ≥1 patient with a grade 5 event) (SAF: N=168) 16](#_Toc191894743)

[**Supplementary Table S12** Second primary malignancies during or after lenalidomide treatment (SAF) 17](#_Toc191894744)

[**Supplementary Table S13** Comparison of subgroup analyses stratified by G8-Geriatric assessment vs. age (>75 vs. ≤75 years) [FAS / SAF] 18](#_Toc191894745)

1. **FIRST-NIS vs. FIRST Trial**

**1.1 Patient characteristics, lenalidomide-treatment, and clinical outcome (total population)**

**Supplementary Table S1** FIRST-NIS vs. FIRST Trial - Patient demographic and clinical characteristics, treatment characteristics, and clinical outcome (total population)

|  | Nückel et al. 2025 FIRST-NIS |  | Benboubker et al. 2014^1^ / Facon et al. 2018^2^  FIRST Trial (Pivotal Phase III) | | |
| --- | --- | --- | --- | --- | --- |
|  | Rd  (N=164) |  | Rdc arm  (N=535) | Rd 18 arm  (N=541) | |
| Baseline patient demographic and clinical characteristics | | | | | |
| Age (years) | | | | | |
| Median (range) | 78 (54, 87) | | 73 (44, 91) | | 73 (40, 89) |
| >75 years [n, (%)] | 116 (71) | | 186 (35) | | 193 (36) |
| ECOG PS [n, (%)] | | | | | |
| 0/1 | 126 (77) | | 412 (77) | 426 (79) | |
| ≥2 | 29 (18) | | 121 (23) | 115 (21) | |
| Unknown/missing | 9 (5) | | 2 (<1) | 0 (0) | |
| ISS stage [n, (%)] | | | | | |
| I/II | 98 (60) | | 319 (60) | 322 (60) | |
| III | 40 (24) | | 216 (40) | 219 (40) | |
| Unknown/missing | 26 (16) | |  |  | |
| CrCl (mL/min) [n, (%)] | | | | | |
| ≥60 | 77 (47) | | 268 (50) | 287 (53) | |
| <60 | 87 (53) | | 267 (50) | 254 (47) | |
| Cytogenetic risk profile^†^ [n/total n, (%)] | | | | | |
| Standard risk | 97/109 (89.0) | | 205/248 (83) | 209/261 (80) | |
| High risk | 12/109 (11.0) | | 43/248 (17) | 52/261 (20) | |
| Lenalidomide treatment duration | | |  |  | |
| Treatment duration (months) | | | | | |
| Median (range)^‡^ | 7.6 (6.3, 9.9) | | 18.4 (0.2, 86.0) | 16.6 (0.2, 23.3) | |
| Number of cycles (n) |  | |  |  | |
| Mean (range) | 11.0 (1, 26)^§^ | | 26.3 (1, 92) | 13.1 (1, 18)^¶^ | |
| Follow-up (months) | | |  |  | |
| Median (range) | 64 (0.2, 94) | | 67 (0, 87) | 67 (0, 87) | |
| Clinical outcome (effectiveness / efficacy) | | |  |  | |
| ORR (≥PR) [n, (%)] | 97 (59) | | 432 (81) | 425 (79) | |
| ≥VGPR [n, %] | 31 (19) | | 260 (49) | 255 (47) | |
| TTR (months) | | | | | |
| Median (95% CI) | 3.1 (2.5, 3.8) | | 1.8 (0.7, 22.2) | 1.8 (0.8, 17.1) | |
| DOR (months) | | | | | |
| Median (95% CI) | 28.4 (21.6, 42.9) | | 35.0 (27.9, 43.4) | 22.1 (20.3, 24.0) | |
| TTF (months) | | | | | |
| Median (95% CI) | n.a. | | 16.9 (14.1, 18.4) | 17.2 (14.6, 18.2) | |
| PFS (months) | | | | | |
| Median (95% CI) | 22.9 (19.3, 28.1) | | 26.0 (20.7, 29.7) | 21.0 (19.7, 22.4) | |
| TTNT (months) | | | | | |
| Median (95% CI) | 29.7 (23.2, 37.5) | | 36.7 (31.9, 45.2) | 28.5 (26.9, 30.4) | |
| OS (months) | | | | | |
| Median (95% CI) | 58.1 (45.7, 71.7) | | 59.1 (53.9, 65.9) | 62.3 (53.6, 58.7) | |

**SUPPLEMENTARY TABLE S1** Data displayed with descriptive statistics [median (range) or frequencies (%)] for the FIRST-NIS (FAS; n = 164) and ITT populations of the Rd continuous (Rdc) and Rd18 treatment arms (FIRST-Trial).

Abbreviations: CI, Confidence Interval; CrCl, Creatinine Clearance; DOR, Duration of Response; ECOG PS, Eastern Cooperative Oncology Group Performance Status; FAS, Full Analysis Set; ISS, International Staging System; N.A., Not Analysed; Mg, Milligram; mL, Millilitre; Min, Minute; ORR, Overall Response Rate; OS, Overall Survival; PFS, Progression-free Survival; PR, Partial Response; Rdc, Lenalidomide plus low-dose dexamethasone until progression; Rd18, Lenalidomide plus low-dose dexamethasone for 18 cycles; SAF, Safety Analysis Set; TTNT, Time to Next (anti-myeloma) Treatment; TTR, Time to Response; TTF, Time to Treatment Failure; VGPR, Very Good Partial Response.

^†^Definition according to pivotal trial^1^: Translocation t(4;14), or t(14;16), or deletion del(17p).

^‡^Ranges represent min-max (FIRST-NIS) and 95% CI (FIRST Trial), respectively.

^§^Number of treatment cycles were documented during the 24-month treatment observation period.

^¶^Number of treatment cycles were limited per study protocol to a maximum of 18 treatment cycles (Rd18 arm).

**1.2 Patient characteristics, lenalidomide-treatment and clinical outcome in patients aged >75 years**

**Supplementary Table S2** FIRST-NIS vs. FIRST Trial - Patient demographic and clinical characteristics, treatment characteristics, and clinical outcome in patients aged older than 75 years

|  | Patients >75 years | | | |
| --- | --- | --- | --- | --- |
|  | Nückel et al. 2025  FIRST-NIS | Hulin et al. 2016^3^; Facon et al. 2018^2^  FIRST Trial (Pivotal Phase III) | | |
|  | Rd  (N=116) | Rdc arm  (N=186) | Rd 18 arm  (N=193) | |
| Baseline patient demographic and clinical characteristics | | | | |
| Age (years) | | | | |
| Median (range) | 79 (75, 91) | 79 (76, 91) | | 79 (76, 89) |
| ECOG PS [n, (%)] | | | | |
| 0/1 | 87 (75.0) | 138 (74) | 148 (77) | |
| ≥2 | 22 (19.0) | 46 (25) | 45 (23) | |
| Missing | 7 (6.0) | 1 (<1) | 0 (0) | |
| ISS stage [n, (%)] | | | | |
| I/II | 61 (53) | 98 (53) | 102 (53) | |
| III | 32 (28) | 88 (47) | 91 (47) | |
| Unknown/missing | 23 (20) | 0 (0) | 0 (0) | |
| CrCl (mL/min) [n, (%)] ^†^ | | | | |
| ≥60 | 41 (35) | n.r. | n.r. | |
| <60 | 75 (64) | n.r. | n.r. | |
| ≥50 | n.a. | 97 (52) | 92 (48) | |
| <50 | n.a. | 89 (48) | 101 (52) | |
| High-risk cytogenetics^‡^ [n, (%)] | 9 (11.5) | 12 (6) | 8 (4) | |
| Lenalidomide treatment duration^§^ | |  |  | |
| Duration (months) | | | | |
| Median (95% CI) | 6.9 (5.0, 8.0) | n.r. | n.r. | |
| Duration (months) | | | | |
| Mean (StD) | n.a. | 20 (16) | 12 (6) | |
| Number of cycles (n) |  |  |  | |
| Mean (StD) | 9.6 (8.2) | n.r. | n.r. | |
| Follow-up (months) | |  |  | |
| Median (range) | 64 (0.2, 94) | 45 (n.r.) | 45 (n.r.) | |
| Clinical outcome (effectiveness / efficacy) | |  |  | |
| ORR (≥PR) [n, (%)] | 63 (54) | 145 (78) | 139 (72) | |
| ≥VGPR [n, (%)] | 20 (17) | 84 (45) | 81 (42) | |
| DOR (months) | | | | |
| Median (95% CI) | 23.5 (16.8, 35.0) | 27 (20, 33) | 21 (17, 23) | |
| PFS (months) | | | | |
| Median (95% CI) | 19.3 (14.2, 25.0) | 20.3 (n.r.) | 19.4 (n.r.) | |
| TTNT (months) | | | | |
| Median (95% CI) | 26.6 (19.3, 37.5) | 39.6 (n.r.) | 26.6 (n.r.) | |
| OS (months)^¶^ | | | | |
| Median (95% CI) | 50.0 (33.7, 68.8) | 48.3 (n.r.) | 45.7 (n.r.) | |

**SUPPLEMENTARY TABLE S2** Data displayed with descriptive statistics [median (range) or frequencies (%)] for the FIRST-NIS (FAS; n = 164) and ITT populations of the Rd continuous (Rdc) and Rd18 treatment arms (FIRST-Trial).

Abbreviations: CI, Confidence Interval; CrCl, Creatinine Clearance; DOR, Duration of Response; ECOG PS, Eastern Cooperative Oncology Group Performance Status; FAS, Full Analysis Set; ISS, International Staging System; N.A., Not Analysed; N.R., Not Reported; Mg, Milligram; mL, Millilitre; Min, Minute; ORR, Overall Response Rate; OS, Overall Survival; PFS, Progression-free Survival; PR, Partial Response; Rdc, Lenalidomide plus low-dose dexamethasone until progression; Rd18, Lenalidomide plus low-dose dexamethasone for 18 cycles; SAF, Safety Analysis Set; StD, Standard Deviation; TTNT, Time to Next (anti-myeloma) Treatment; VGPR, Very Good Partial Response.

^†^In the FIRST-NIS, renal impairment (thresholds) was defined according to the initial publication of the pivotal trial.

^‡^Definition according to pivotal trial: Translocation t(4;14), or t(14;16), or deletion del(17p).

^§^Median duration of lenalidomide-treatment was not reported for the FIRST Trial; mean treatment duration was not analysed in the FIRST-NIS.

^¶^Median OS in the FIRST Trial were analysed with a median Follow-up of 67 months.

**1.3 G8-Geriatric assessment tool vs. Simplified frailty Index**

##### 1.3.1 Selected patient demographic, clinical and treatment characteristics

- In our NIS, the treating physicians used the G8-Geriatric assessment tool ^4,5^ at baseline to further characterize the patient’s individual health status (impairment status).
- Based on the clinical data from the FIRST trial, Facon et al ^6^ retrospectively developed a new simplified frailty score using scores for age, comorbidities (CCI), and ECOG PS.

**Supplementary Table S3** FIRST-NIS vs. FIRST Trial - Patient demographic, clinical and treatment characteristics stratified by G8-geriatric assessment (impairment status) and simplified frailty index (frailty status)

|  | Nückel et al. 2025  FIRST-NIS | | |  | Facon et al. 2019^6^  FIRST Trial (Pivotal Phase III) | | | |
| --- | --- | --- | --- | --- | --- | --- | --- | --- |
|  | G8-Geriatric  assessment tool^†^ | | |  | Simplified  frailty scale^‡^ | | | |
|  | Rd  (N=164) | | |  | Rdc + Rd18  (N=1618) | | | |
|  | Impaired  (N=86) | Non-impaired  (N=33) | Missing  (N=45) |  | Frail  (N=790) | | Non-frail  (N=828) | |
| Selected baseline patient demographic characteristics | | | | | | | | |
| Age (years) | | | | | | | | |
| Median (range) | 78.8 | 76.4 | 77.5 | | 77 (44, 92) | | 70 (40, 80) | |
| >75 years [n, (%)] | 65 (75.6) | 23 (69.7) | 28 (62.2) | | 491 (62.1) | | 74 (9.0) | |
| Comorbidities [n, (%)] |  |  |  | |  |  |  |  |
| 0/1 | 14 (10.5) | 11 (33.3) | 9 (20.0) | | n.r. | | n.r. | |
| ≥2 | 77 (89.5) | 22 (66.7) | 36 (80.0) | | n.r. | | n.r. | |
|  |  |  |  | |  | |  | |
| ECOG PS [n, (%)] |  |  |  | |  |  |  |  |
| 0/1 | 62 (72.1) | 33 (100.0) | 31 (68.9) | | 441 (55.8) | | 828 (100.0) | |
| ≥2 | 20 (23.2) | 0 (0) | 9 (20.0) | | 349 (44.2) | | 0 (0) | |
| Unknown/missing | 4 (4.7) | 0 (0) | 5 (11.1) | | 0 (0) | | 0 (0) | |
| CrCl (mL/min) [n, (%)] |  |  |  | |  |  |  |  |
| ≥60 | 32 (37.2) | 22 (66.7) | 23 (48.9) | | 312 (39) | | 529 (64) | |
| <60 | 54 (62.8) | 11 (33.3) | 22 (51.1) | | 478 (61) | | 299 (36) | |
| ISS Stage [n, (%)] |  |  |  | |  |  |  |  |
| I/II | 50 (58.1) | 23 (69.7) | 25 (55.6) | | 419 (53) | | 575 (69) | |
| III | 27 (31.4) | 8 (24.2) | 5 (11.1) | | 371 (47) | | 253 (31) | |
| Unknown/missing | 9 (10.5) | 2 (6.1) | 15 (33.3) | | 0 (0) | | 0 (0) | |
| Lenalidomide treatment characteristics^§^ | |  |  | |  | |  | |
| Dose / day of administration (mg) | | |  | |  | |  | |
| Median | 15.3 | 20.7 | 17.7 | | n.r. | | n.r. | |
| Range | (4.3, 25.0) | (6.4, 25.0) | (7.7, 25.0) | |  | |  | |

**SUPPLEMENTARY TABLE S3** Data displayed with descriptive statistics [median (range) or frequencies (%)] for the FIRST-NIS (FAS; n = 164) and ITT populations of the pooled Rd continuous (Rdc) and Rd18 treatment arms stratified by frailty status (FIRST-Trial).

Abbreviations: CI, Confidence Interval; CrCl, Creatinine Clearance; ECOG PS, Eastern Cooperative Oncology Group Performance Status FAS, Full Analysis Set; ITT, Intent to Treat; G8-GA, G8-Geriatric assessment; ISS, International Staging System; N.R., Not Reported; Mg, Milligram; mL, Millilitre; Min, Minute; Rdc, Lenalidomide plus low-dose dexamethasone until progression; Rd18, Lenalidomide plus low-dose dexamethasone for 18 cycles; SAF, Safety Analysis Set.

^†^The validated G8-Geriatric assessment tool was used to assess the individual health status of the patient; impaired patient status is defined as G8-GA score ≤14, and non-impaired patient status is defined as G8-GA score >14, respectively).

^‡^Patient frailty status was defined by Facon et al 2019 using age, comorbidities (CCI), and ECOG-PS.

^§^FIRST-NIS treatment duration was analysed in the SAF (N = 168): impaired: N = 89; non-impaired: N = 34; missing: N = 45; lenalidomide daily dose was defined as the cumulative dose divided by dose exposure (mg/day).

##### 1.3.2 Clinical outcome (effectiveness / efficacy)

**Supplementary Table S4** FIRST-NIS vs. FIRST Trial - Clinical outcome stratified by G8-Geriatric assessment (impairment status) and simplified frailty index (frailty status)

|  | Nückel et al. 2025  FIRST-NIS | | | |  | Facon et al. 2019^6^  FIRST Trial (Pivotal Phase III) | | | |
| --- | --- | --- | --- | --- | --- | --- | --- | --- | --- |
|  | G8-Geriatric  assessment tool^†^ | | | |  | Simplified  frailty scale^‡^ | | | |
|  | Rd  (N=164) | | | |  | Rdc arm  (N=533) | | Rd18 arm  (N=541) | |
|  | Impaired  (N=86) | Non-impaired  (N=33) | | Missing  (N=45) |  | Frail  (N=266) | Non-frail  (N=267) | Frail  (N=267) | Non-frail  (N=274) |
| Impairment / frailty status | | | | | | | | | |
| Proportion (%) | 52.4 | 20.1 | | 27.4 | | 49.9 | 50.1 | 49.4 | 50.6 |
| ORR^§^ | | | | | | | | | |
| n, (%) | 46 (53.5) | 22 (66.7) | | 29 (64.4) | | n.r. | n.r. | n.r. | n.r. |
| (95%-CI) | (43.0, 63.7) | (49.5, 80.3) | (49.8, 76.8) | | |  |  |  |  |
| PFS (months) | | | | | | | | | |
| Median | 19.3 | 45.6 | | 25.4 | | 19.4 | 31.3 | 19.4 | 22.1 |
| (95% CI) | (13.7, 25.2) | (19.3, 66.5) | | (18.6, 33.8) | | n.r. | n.r. | n.r. | n.r. |
| OS (months) | | | | | | | | | |
| Median | 44.1 | 84.6 | | 65.8 | | 44.3 | 75.2 | 41.8 | 80.1 |
| (95% CI) | (27.2, 60.1) | (52.5, NA) | | (31.8, NA) | | n.r. | n.r. | n.r. | n.r. |

**SUPPLEMENTARY TABLE S4** Data displayed with descriptive statistics [median (range) or frequencies (%)] for the FIRST-NIS (FAS; n = 164) and ITT populations of the Rd continuous (Rdc) and Rd18 treatment arms stratified by frailty status (FIRST-Trial).

Abbreviations: CI, Confidence Interval; FAS, Full Analysis Set; N.R., Not Reported; ORR, Overall Response Rate; OS, Overall Survival; PFS, Progression-free Survival; Rdc, Lenalidomide plus low-dose dexamethasone until progression; Rd18, Lenalidomide plus low-dose dexamethasone for 18 cycles.

^†^The validated G8-Geriatric assessment (G8-GA) tool was used to assess the individual health status of the patient; impaired patient status is defined as G8-GA score ≤14, and non-impaired patient status is defined as G8-GA score >14, respectively).

^‡^Patient frailty status was defined by Facon et al 2019 using age, comorbidities (Charlson Comorbidity Index), and ECOG PS.

^§^In the pivotal trial, ORRs stratified by frailty status were reported as pooled data only (Rdc + Rd18 treatment arms): ORR (frail patients

vs. non-frail patients): 72% vs. 79%, respectively).

1. **Methods**

**2.1 Definition of secondary effectiveness endpoints**

- Progression-free survival (PFS) was defined as the time interval between first intake of lenalidomide (R) until progressive disease (PD), or death (whichever came first).
- Time to response (TTR) was defined as the time interval from first intake of R to the first occurrence of a response [≥ partial response (PR)].
- Duration of response (DOR) was defined as the time interval from first documented response (≥ PR) to a documented PD or death due to PD.
- Time to second-line anti-myeloma therapy (TT2^nd^-AMT) was defined as the time interval from first intake of R to onset of the TT2^nd^-AMT.
- Overall survival (OS) was defined as the time interval from first intake of R until death due to any cause.

1. **Renal impairment – Patient and treatment characteristics**

**3.1 Renal impairment - Selected patient and treatment characteristics stratified by CrCl (mL/min)**

- The median lenalidomide treatment duration was shorter in the subgroup of patients with severe and moderate renal impairment (RI) compared to the subgroup of patients with mild or no RI).
- Patients with severe RI were documented with the lowest dose intensity per day of administration (10.0 mg/day) compared to the patients with moderate (15.4 mg/day) and mild RI (18.1 mg/day), or normal renal function (18.1 mg/day).
- Discontinuation of R treatment due to toxicity was similar in the subgroups stratified by renal impairment.

**Supplementary Table S5** Renal impairment – selected patient and treatment characteristics stratified by creatinine clearance

|  | SAF (N=168) | | | | | |  |
| --- | --- | --- | --- | --- | --- | --- | --- |
|  | CrCl  <30 mL/min  Severe RI  (N=16) | | CrCl  30-<60 mL/min  Moderate RI  (N=72) | CrCl  60-<90 mL/min  Mild RI  (N=62) | | CrCl  ≥90 mL/min  No RI  (N=18) |  |
| Patient characteristic^†^ |  | |  |  | |  | |
| Age (years) |  | |  |  | |  | |
| Median (range) | 84.2 (67.3, 88.4) | | 79.0 (67.9, 91.3) | 76.2 (64.2, 84.0) | | 72.3 (53.6, 80.7) | |
| ISS stage [n, (%)] |  | |  |  | |  | |
| I/II | 5 (31.2) | | 34 (47.9) | 45 (75.0) | | 14 (82.3) | |
| III | 7 (43.8) | | 22 (31.0) | 9 (15.0) | | 2 (11.8) | |
| Missing/unknown | 4 (25.0) | | 15 (21.1) | 6 (10.0) | | 1 (5.9) | |
| Lenalidomide treatment characteristics | | | | |  | |  |
| Starting dose [n, (%)] | | | | |  | |  |
| 25mg | 5 (31.3) | | 35 (48.6) | 41 (66.1) | | 11 (61.1) | |
| <25mg | 11 (68.8) | | 37 (51.4) | 21 (33.9) | | 7 (38.9) | |
| Treatment duration (months) | | | | |  | |  |
| Median (95% CI) | 2.0 (0.6, 4.5) | | 7.5 (5.3, 9.9) | 9.8 (6.9, 16.2) | | 12.6 (5.6, 35.2) | |
| Number of cycles (n) | | | | |  | |  |
| Median (min-max) | 2.0 (1, 26) | | 7.5 (1, 26) | 9.5 (1, 26) | | 13.5 (1, 26) | |
| Mean (StD) | 3.8 (3.9) | | 10.6 (8.6) | 12.4 (9.3) | | 14.1 (9.7) | |
| Dose / day of administration^§^ (mg) | | | | |  | |  |
| Median (min-max) | 10.0 (4.3, 25.0) | | 15.4 (5.1, 25.0) | 18.1 (5.9, 25.0) | | 18.1 (7.5, 25.0) | |
| Treatment modifications due to AE [n, (%)] |  | |  |  | |  |  |
| Any study drug |  | |  |  | |  |  |
| Interruption | 7 (43.8) | | 42 (58.3) | 38 (61.3) | | 11 (61.1) |  |
| Dose reduction | 1 (6.3) | | 19 (26.4) | 25 (40.3) | | 7 (38.9) |  |
| Discontinuation | 9 (56.3) | | 36 (50.0) | 21 (33.9) | | 8 (44.4) |  |
| Lenalidomide | |  |  |  | |  |  |
| Discontinuation due to toxicity | | 5 (31.3) | 24 (33.3) | 15 (24.2) | | 6 (33.3) |  |

**SUPPLEMENTARY TABLE S5** Data stratified by CrCl displayed with descriptive statistics [median (range) or frequencies (%)] for the SAF (n = 168).

Abbreviations: AE, Adverse Event; CI, Confidence Interval; CrCl, Creatinine Clearance; ISS, International Staging System; N/n, Number; Max, Maximum; Min, Minimum; mL, Millilitre; Mg, Milligram; RI, Renal Impairment; SAF, Safety Analysis Set; StD, Standard Deviation.

Duration of lenalidomide therapy was calculated as the time interval from first intake of lenalidomide until last intake of lenalidomide during the observation period plus min {7*((1 + last intake last cycle – first intake last cycle) / 21), 7} as a correction term for the therapy-free interval at the end of a cycle.

^†^Patient characteristics were analysed in the FAS (N = 164; severe RI (N = 16); moderate RI (N = 71); mild RI (N = 60); no RI (N = 17)).

^‡^A starting dose of 25mg lenalidomide was assumed for all patients when calculating the relative dose intensity.

^§^Lenalidomide daily dose was defined as the cumulative dose divided by dose exposure (mg/day).

1. **Multivariate COX regression analysis**

**4.1 Covariates as predictors for PFS**

- Multivariate Cox regression analysis with pre-defined covariates as potential predictors for PFS (n=57/159 censored cases) suggested statistically significant improved outcome in patients with better renal function (per increase of CrCl value by 10ml/min steps: HR=0.85; 95% CI 0.77-0.93; p<0.001), and higher pre-treatment level of hemoglobin (Hb) (per increase of Hb level by 1g/dl steps: HR=0.89; 95% CI 0.8-0.98; p=0.016).
- A higher ECOG performance status at baseline (≥2 vs. 0/1) was observed as a negative predictor of PFS (HR=2.72; 95% CI: 1.64-4.54; p<0.001).
- The corresponding median age, median PFS and median OS stratified by the covariates are presented in the supplementary Table S7.

**Supplementary Table S6** Multivariate Cox regression analysis

|  | Hazard Ratio  (HR) | | 95% CI  (HR) | p-value |
| --- | --- | --- | --- | --- |
| Covariate | | | | |
| CrCl (per 10 ml/min) | | 0.85 | (0.77 - 0.93) | <0.001*** |
| ECOG PS: Missing vs. 0/1 | | 0.81 | (0.35 - 1.88) | 0.621 |
| ECOG PS: ≥2 vs. 0/1 | | 2.72 | (1.64 - 4.54) | <0.001*** |
| Hb level (per 1 g/dl) | | 0.89 | (0.81 - 0.98) | 0.016* |

**SUPPLEMENTARY TABLE S6** Multivariate Cox regression analysis with pre-defined covariates as potential predictors for PFS.

Abbreviations: CI, Confidence Interval; CrCl, Creatinine Clearance; dL, Decilitre; ECOG PS, Eastern Cooperative Oncology Group Performance Status; G, Gram; Hb, Hemoglobin; HR, Hazard Ration; mL, Millilitre; Min, Minute; PFS, Progression-free Survival

Estimates of hazard ratio and confidence intervals were obtained using a multivariate Cox proportional hazard regression model. The model was build using backward selection. Reported p-values had an exploratory character.

Pre-specified variables (all measured at baseline) were initially selected by medical experts as potentially influencing variables: “Renal function (CrCL)”, “Hemoglobin level”, “Age at date of informed consent”, “Gender”, “ECOG Performance Status”, “Number of comorbidities”, “Bone marrow plasma cell percentage”, “International Staging system (ISS) stage”, “Diabetes”, “Smoking status”, “Cardiac disorder”, “Cerebrovascular disorder”, “(Peripheral) vascular disorder”, “Arterial hypertension at baseline (binary)”, “Development or worsening of hypertension”. The variables “Bone marrow plasma cell percentage” and “Development or worsening of hypertension” were excluded from the original model because of >90% missing values and because of <5% events, respectively. Variables removed in backward selection procedure were. “Diabetes”, “Age at date of informed consent”, “Gender”, “Number of comorbidities”, “Smoking status”, ”Cardiac disorder”, “Cerebrovascular disorder”, “(Peripheral) vascular disorder”, “Arterial hypertension”, and “ISS stage”.

Variables remaining in the model as predictors for PFS were: “Creatinine Clearance”, “ECOG Performance Status”, and “Hemoglobin level”. For “Creatinine clearance (per 10ml/min)”, the gradual increase of the CrCl values by 10ml/min was used as reference levels. For “Hemoglobin level (per 1g/dl)”, the gradual increase of the Hb values by 1g/dl was used as reference levels. For “ECOG Performance Status “missing vs. 0/1” and “ECOG Performance Status ≥2 vs. 0/1”, ECOG Performance Status 0/1 was used as reference value, respectively.

159 patients were used for the calculation of the regression model. 5 patients were excluded due to missing values. Of the 159 patients included in the model, 102 had an event, while 57 (35.8%) did not had an event and were censored. Model fit statistics: Global Likelihood Ratio test: p-value: <.0001; Convergence criterion (GCONV=1E-8) was satisfied, Efron method was used to control for ties.

P-values <0.001 are marked with “***”, p-values <0.01 are marked with “**” and p-values <0.05 are marked with “*”.

**4.2 PFS and OS stratified by ECOG PS, creatinine clearance, and hemoglobin at baseline**

- Patients presented with an ECOG PS of 0/1 at baseline had markedly longer median PFS and median OS compared to those presented with an ECOG PS of ≥2 (95% confidence intervals (95% CI) were not overlapping).
- Patients presented with no or mild renal impairment (RI) at baseline (CrCl >90 and 60 - <90 mL/min, respectively) were younger and had longer median PFS and OS compared to patients with moderate or severe RI (CrCl <30 and 30 - <60 mL/min, respectively).
- Patients presented with hemoglobin level (Hb) of ≥10 g/dl at baseline had markedly longer median PFS and median OS compared to those presented with Hb of <10 g/dl (95% CIs were not overlapping).

**Supplementary Table S7** Progression-free survival and overall survival stratified by ECOG PS, CrCl, and hemoglobin at baseline

| Subgroup | |  | Median age  [years]  (range) | FAS  [n, (%)]  N=164 | Events  [n, (%)] | Median PFS [months]  (95% CI) | Events  [n, (%)] | Median OS [months]  (95% CI) |
| --- | --- | --- | --- | --- | --- | --- | --- | --- |
| ECOG PS | | | | | | | | |
|  | 0/1 |  | 77.6 (53.6, 91.3) | 126 (76.8) | 78 (61.9) | 25.0 (20.8, 32.9) | 60 (47.6) | 65.8 (49.9, 84.6) |
|  | ≥2 |  | 77.0 (65.3, 88.8) | 29 (17.7) | 21 (72.4) | 10.2 (7.3, 15.5) | 20 (69.0) | 14.6 (8.3, 48.0) |
|  | Missing |  | 80.4 (69.7, 88.4) | 9 (5.5) | 6 (66.7) | 44.1 (1.6, NA) | 5 (55.6) | 68.8 (1.6, NA) |
| CrCl (mL/min) | | | | | | | | |
|  | <30 |  | 84.2 (67.3, 88.4) | 16 (9.8) | 12 (75.0) | 4.4 (1.6, 23.0) | 10 (62.5) | 8.8 (1.6, NA) |
|  | 30 - <60 |  | 79.0 (67.9, 91.3) | 71 (43.3) | 51 (71.8) | 19.4 (15.5, 25.4) | 42 (59.2) | 46.2 (30.3, 68.8) |
|  | 60 - <90 |  | 76.2 (64.2, 84.0) | 60 (36.6) | 33 (55.0) | 28.1 (21.6, 50.0) | 26 (43.3) | 84.2 (52.5, NA) |
|  | ≥90 |  | 72.3 (53.6, 80.7) | 17 (10.4) | 9 (52.9) | 48.0 (10.8, NA) | 7 (41.2) | NA (47.7, NA) |
| Hemoglobin (g/dl) | | | | | | |  |  |
|  | ≥10 |  | 77.2 (53.6, 91.3) | 99 (60.4) | 58 (58.6) | 31.5 (22.9, 46.3) | 41 (41.4) | 71.7 (60.1, NA) |
|  | <10 |  | 78.9 (62.7, 88.8) | 65 (39.6) | 47 (72.3) | 18.6 (9.9, 22.2) | 44 (67.7) | 33.0 (22.0, 48.0) |

**SUPPLEMENTARY TABLE S7** Data displayed with descriptive statistics [median (range) or frequencies (%)] stratified by ECOG PS, CrCl, and hemoglobin at baseline (FAS).

Abbreviations: CI, Confidence Interval; CrCl, Creatinine Clearance; dL, Decilitre; ECOG PS, Eastern Cooperative Oncology Group Performance Status; FAS = Full Analysis Set; G, Gram; Hb, Hemoglobin; Mg, Milligram; mL, Millilitre; Min, Minute; OS, Overall Survival; PFS, Progression-Free Survival

1. **Health-related Quality of Life (QoL)**

**5.1 Return and evaluability of patient-reported QoL questionnaires**

**Supplementary Table S8** Return and evaluability of questionnaires

| Time point | Patients  on-treatment | Questionnaires  evaluable† | |
| --- | --- | --- | --- |
|  | FAS (N=164) | N | % |
| Baseline (T0) | 164 | 142 | 86.6 |
| 3 months (T1) | 126 | 92 | 73.0 |
| 6 months (T2) | 94 | 73 | 77.7 |
| 12 months (T3) | 59 | 49 | 83.0 |
| 18 months (T4) | 45 | 41 | 91.1 |
| 24 months (T5) | 12 | 8 | 66.7 |

**SUPPLEMENTARY TABLE S8** Return of questionnaires.

Abbreviations: FAS = Full Analysis Set; N, Number; T, Time.

^†^A questionnaire was considered “evaluable” if at least one of the relevant scores could be calculated and the patient filled in the questionnaire under study treatment.

**5.2 Changes from baseline in patient-reported quality of life (QoL) questionnaire score**

**Supplementary Table S9** Changes from baseline in patient-reported quality of life (QoL) questionnaire score

| Questionnaire /  Subscale^†^ / Timepoint | N | Mean | StD |
| --- | --- | --- | --- |
| EORTC QLQ C-30 |  |  |  |
| Global Health status / Quality of life |  |  |  |
| 3 months (T1) | 74 | 0.2 | 22.5 |
| 6 months (T2) | 58 | 2.4 | 23.0 |
| 12 months (T3) | 39 | 1.5 | 21.2 |
| 18 months (T4) | 33 | -1.0 | 24.6 |
| 24 months (T5) | 8 | -1.0 | 9.4 |
| Physical function |  |  |  |
| 3 months (T1) | 78 | -6.0^+^ | 19.3 |
| 6 months (T2) | 61 | -2.7 | 19.1 |
| 12 months (T3) | 38 | -0.2 | 22.0 |
| 18 months (T4) | 33 | -4.4 | 25.6 |
| 24 months (T5) | 8 | -8.3^+^ | 22.5 |
| Fatigue |  |  |  |
| 3 months (T1) | 77 | 6.6^+^ | 23.2 |
| 6 months (T2) | 61 | 3.2 | 23.3 |
| 12 months (T3) | 39 | 3.8 | 25.4 |
| 18 months (T4) | 33 | 8.8^+^ | 22.4 |
| 24 months (T5) | 8 | 12.5^++^ | 22.6 |
| Pain |  |  |  |
| 3 months (T1) | 77 | -0.2 | 31.5 |
| 6 months (T2) | 61 | -3.8 | 35.6 |
| 12 months (T3) | 39 | -9.8^+^ | 32.0 |
| 18 months (T4) | 33 | -2.5 | 25.0 |
| 24 months (T5) | 8 | 6.3^+^ | 21.7 |
| EORTC QLQ-MY 20 |  | |  |
| Disease symptoms |  |  |  |
| 3 months (T1) | 72 | 0.6 | 25.4 |
| 6 months (T2) | 57 | -3.8 | 24.5 |
| 12 months (T3) | 38 | -3.9 | 26.1 |
| 18 months (T4) | 31 | 2.3 | 22.6 |
| 24 months (T5) | 7 | 4.9 | 16.2 |
| Side effects of treatment |  |  |  |
| 3 months (T1) | 76 | 8.9 | 16.0 |
| 6 months (T2) | 61 | 5.5 | 16.5 |
| 12 months (T3) | 40 | 4.6 | 13.6 |
| 18 months (T4) | 33 | 6.4 | 13.5 |
| 24 months (T5) | 8 | 14.0^+^ | 9.9 |

**SUPPLEMENTARY TABLE S9** Changes from baseline in six clinically relevant patient-reported quality of life (QoL) questionnaire scores. Data displayed with numbers of observations, mean and standard deviation (StD). Higher scores in the global health status/QoL and functional scales (physical function) correspond to higher perceived QoL/ healthy level of functioning, while higher scores in the symptom scales (fatigue, pain, disease symptoms, side effects of treatment) correspond to higher level of perceived symptoms severity based on the questionnaire results.

Abbreviations: EORTC, European Organisation For Research And Treatment Of Cancer; HRQoL, Health-related Quality-of-life; N, Number; StD, Standard deviation; T, Time.

T0: Baseline; T1: Month 3; T2: Month 6; T3: Month 12; T4: Month 18; T5: Month 24 (after start of Rd treatment).

EORTC QLQ-C30 and QLQ-MY20 total score range from 1 – 100.

^†^Clinically relevant HRQoL domains as previously reported for the pivotal trial.^7^

Minimal important difference (MID) thresholds according to the established definition of MID for EORTC QLQ-C30^8^ and EORTC-MY20:^9^

Global Health Status: small deterioration -5 to -10

Physical Function: small deterioration -5 to -10

Pain: small deterioration -3 to -11; small improvement +5 to +9; medium improvement +9 to +14

Fatigue: small deterioration -5 to -10; medium deterioration -10 to -15

Disease Symptoms: deterioration -10

Side Effects of Treatment: deterioration -10

“+” and “++” indicate mean value outside of the proposed range of MID (“small” and “medium deterioration”, respectively).

1. **Adverse events**

**6.1 Adverse events (all grades)**

- In the SAF (n=168), 165 (98.2%) patients were reported with any treatment-emergent AE.
- Most common AE (≥10%, any grade) were anaemia (29.8%), thrombocytopenia (20.8%), diarrhoea (20.8%), fatigue (18.5%), back pain (14.9%), dyspnoea (11.3%), rash (10.7%), and constipation (10.1%).
- The most common (≥3%) AEs of grade 3/4 were anaemia (8.3%), thrombocytopenia (7.1%), dyspnoea (4.8%), pneumonia (4.8%), neutropenia (3.6%), back pain (3.6%), bone pain (3.6%), and acute kidney injury (3.0%).
- Overall, 96 (57.1%) patients were reported with at least one serious adverse event (SAE), where pneumonia (n=11; 6.5%) was the most frequently documented event.

**Supplementary Table S10** Treatment-emergent adverse events (AEs) occurring in >5% (any grade) of the safety population (SAF: N=168)

| Adverse event, n (%) | Any event | Grade 1/2 | Grade 3/4 | Grade 5 |
| --- | --- | --- | --- | --- |
| Any event (Preferred Term) | 165 (98.2) | 145 (86.3) | 108 (64.3) | 15 (8.9) |
| Hematologic adverse events | | | | |
| Anemia | 50 (29.8) | 44 (26.2) | 14 (8.3) |  |
| Thrombocytopenia^†^ | 35 (20.8) | 27 (16.1) | 12 (7.1) |  |
| Leukopenia | 15 (8.9) | 13 (7.7) | 2 (1.2) |  |
| Neutropenia | 14 (8.3) | 10 (6.0) | 6 (3.6) |  |
| Nonhematologic adverse events | | | | |
| Diarrhoea | 35 (20.8) | 32 (19.0) | 4 (2.4) |  |
| Fatigue | 31 (18.5) | 29 (17.3) | 2 (1.2) |  |
| Back pain | 25 (14.9) | 19 (11.3) | 6 (3.6) |  |
| Dyspnoea | 19 (11.3) | 12 (7.1) | 8 (4.8) |  |
| Rash | 18 (10.7) | 17 (10.1) | 1 (0.6) |  |
| Constipation | 17 (10.1) | 16 (9.5) | 1 (0.6) |  |
| Pneumonia | 16 (9.5) | 6 (3.6) | 8 (4.8) | 2 (1.2) |
| General physical health deterioration | 15 (8.9) | 10 (6.0) | 4 (2.4) | 2 (1.2) |
| Nausea | 15 (8.9) | 15 (8.9) |  |  |
| Oedema peripheral | 15 (8.9) | 13 (7.7) | 3 (1.8) |  |
| Blood creatinine increased | 14 (8.3) | 14 (8.3) | 1 (0.6) |  |
| Hypocalcaemia | 14 (8.3) | 14 (8.3) |  |  |
| Bronchitis | 13 (7.7) | 11 (6.5) | 3 (1.8) |  |
| Decreased appetite | 13 (7.7) | 13 (7.7) |  |  |
| Dizziness | 13 (7.7) | 13 (7.7) |  |  |
| Vertigo | 13 (7.7) | 11 (6.5) | 2 (1.2) |  |
| Bone pain | 12 (7.1) | 11 (6.5) | 6 (3.6) |  |
| Muscle spasms | 11 (6.5) | 11 (6.5) |  |  |
| Polyneuropathy | 11 (6.5) | 10 (6.0) | 2 (1.2) |  |
| Nasopharyngitis | 10 (6.0) | 10 (6.0) |  |  |
| Pruritus | 10 (6.0) | 10 (6.0) |  |  |
| Acute kidney injury | 9 (5.4) | 1 (0.6) | 5 (3.0) | 3 (1.8) |
| Arthralgia | 9 (5.4) | 7 (4.2) | 2 (1.2) |  |
| Insomnia | 9 (5.4) | 9 (5.4) |  |  |

**SUPPLEMENTARY TABLE S10** Data displayed with descriptive statistics [frequencies (%)] for the SAF (n=168).

Abbreviations: AE, Adverse Event; CTCAE, Common Terminology Criteria for Adverse Events; MedDRA, Medical Dictionary for Regulatory Activities; SAF, Safety Analysis Set.

Treatment-emergent adverse events were graded according to the National Cancer Institute Common Terminology Criteria for AEs (NCI CTCAE version 4.03) and classified to preferred terms (PT) according to the Medical Dictionary for Regulatory Activities (MedDRA version 26.0).

^†^“Thrombocytopenia” combined AE Term included the following MedDRA PTs: Thrombocytopenia, Platelet count decreased.

**6.2 Adverse events of specific interest**

**Supplementary Table S11** Selected treatment-emergent adverse events (AEs) in ≥1% of patients with either grade 3/4 events or ≥1 patient with grade 5 event) (SAF: N=168)

| Adverse event (Preferred Term), n (%) | Any event | Grade 1/2 | Grade 3/4 | Grade 5 |
| --- | --- | --- | --- | --- |
| Hematologic events^†^ | | | | |
| Any event | 78 (46.4) | 68 (40.5) | 28 (16.7) |  |
| Anemia | 50 (29.8) | 44 (26.2) | 14 (8.3) |  |
| Thrombocytopenia | 21 (12.5) | 15 (8.9) | 8 (4.8) |  |
| Leukopenia | 15 (8.9) | 13 (7.7) | 2 (1.2) |  |
| Platelet count decreased | 14 (8.3) | 12 (7.1) | 4 (2.4) |  |
| Neutropenia | 14 (8.3) | 10 (6.0) | 6 (3.6) |  |
| Neutrophil count decreased | 5 (3.0) | 4 (2.4) | 2 (1.2) |  |
| Infections^‡^ | | | | |
| Any event | 68 (40.5) | 48 (28.6) | 27 (16.1) | 3 (1.8) |
| Pneumonia | 16 (9.5) | 6 (3.6) | 8 (4.8) | 2 (1.2) |
| Bronchitis | 13 (7.7) | 11 (6.5) | 3 (1.8) |  |
| Urinary tract infection | 5 (3.0) | 1 (0.6) | 4 (2.4) |  |
| Cystitis | 4 (2.4) | 2 (1.2) | 2 (1.2) |  |
| Pulmonary sepsis | 1 (0.6) | 0 (0.0) | 0 (0.0) | 1 (0.6) |
| Cardiac events^§^ |  |  |  |  |
| Any event | 28 (16.7) | 12 (7.1) | 18 (10.7) | 2 (1.2) |
| Atrial fibrillation | 5 (3.0) | 2 (1.2) | 3 (1.8) |  |
| Cardiac failure | 5 (3.0) | 1 (0.6) | 4 (2.4) |  |
| Acute myocardial infarction | 3 (1.8) | 0 (0.0) | 3 (1.8) |  |
| Coronary artery disease | 2 (1.2) | 0 (0.0) | 2 (1.2) |  |
| Cardiac failure chronic | 2 (1.2) | 0 (0.0) | 1 (0.6) | 1 (0.6) |
| Cardiac arrest | 1 (0.6) | 0 (0.0) | 0 (0.0) | 1 (0.6) |
| Fatigue^¶^ |  |  |  |  |
| Any event | 38 (22.6) | 36 (21.4) | 2 (1.2) |  |
| (Peripheral) vascular events^○^ |  |  |  |  |
| Any event | 14 (8.3) | 6 (3.6) | 7 (4.2) | 1 (0.6) |
| Deep vein thrombosis | 5 (3.0) | 3 (1.8) | 2 (1.2) |  |
| Pulmonary embolism | 5 (3.0) | 0 (0.0) | 4 (2.4) | 1 (0.6) |
| Polyneuropathy^◊^ |  |  |  |  |
| Any event | 18 (10.7) | 16 (9.5) | 3 (1.8) |  |
| Polyneuropathy | 11 (6.5) | 10 (6.0) | 2 (1.2) |  |
| Cataract |  |  |  |  |
| Any event | 2 (1.2) | 0 (0.0) | 2 (1.2) |  |

**SUPPLEMENTARY TABLE S11** Data displayed with descriptive statistics [frequencies (%)] for the SAF (n=168).

Abbreviations: AE, Adverse Event; CTCAE, Common Terminology Criteria for Adverse Events; MedDRA, Medical Dictionary for Regulatory Activities; SAF, Safety Analysis Set.

Treatment-emergent adverse events were graded according to the National Cancer Institute Common Terminology Criteria for AEs (NCI CTCAE version 4.03) and classified to system organ class (SOC) preferred terms (PT) according to the Medical Dictionary for Regulatory Activities (MedDRA version 26.0). The table is sorted by frequencies of grade 3/4 events within the pooled adverse event categories.

^†^Hematologic adverse events were defined as SOC “blood and lymphatic system disorders” and PTs “haemoglobin decreased”, “neutrophil count decreased”, “platelet count decreased”, “white blood cell count decreased”, and “bone marrow band”.

^‡^Infections were defined by SOC “infections and infestations” and PT “influenza A virus test positive”.

^§^Cardiac disorders were defined as SOC “cardiac disorders” and HLT “cardiac valve therapeutic procedures”, PTs “congenital aortic valve stenosis”, and “coronary arterial stent insertion”.

^¶^Fatigue was defined as PTs “asthenia”, “fatigue”, and “chronic fatigue syndrome”.

^○^Peripheral vascular disorders were defied as PTs “pulmonary embolism”, “deep vein thrombosis” and HLTs “peripheral embolism and thrombosis”, “non-site specific embolism and thrombosis”.

^◊^Polyneuropathy was defined as PT “paraesthesia” and HLGT “peripheral neuropathies”.

**6.2 Second primary malignancies**

**Supplementary Table S12** Second primary malignancies during or after lenalidomide treatment (SAF)

| MedDRA  System organ class | MedDRA  Preferred Term | Start SPM  on-/post-treatment |  |  |  | SAF (N=168) |
| --- | --- | --- | --- | --- | --- | --- |
| Neoplasms benign, malignant and  unspecified (incl cysts and polyps) [n, %] | | | | | | |
| Any patient^†^ |  |  |  |  |  | 12 (7.1) |
| Haematologic tumour | |  |  |  |  |  |
|  | Any event |  |  |  |  | 2 (1.2) |
|  | Myelodysplastic syndrome^‡^ | Post-treatment |  |  |  | 1 (0.6) |
|  | Acute myeloid leukaemia^‡^ | Post-treatment |  |  |  | 1 (0.6) |
| Solid tumour |  |  |  |  |  |  |
|  | Any event |  |  |  |  | 13 (7.7) |
|  | Bowen's disease^§^ | On-treatment |  |  |  | 1 (0.6) |
|  | Squamous cell carcinoma of skin^§^ | On-treatment |  |  |  | 1 (0.6) |
|  | Gastrointestinal neoplasm | On-treatment |  |  |  | 1 (0.6) |
|  | Lung neoplasm malignant | On-treatment |  |  |  | 1 (0.6) |
|  | Malignant melanoma | Post-treatment |  |  |  | 1 (0.6) |
|  | Metastasis^≠^ | Post-treatment |  |  |  | 1 (0.6) |
|  | Pancreatic neoplasm | On-treatment |  |  |  | 1 (0.6) |
|  | Prostate cancer^¶^ | Post-treatment^#^ |  |  |  | 1 (0.6) |
|  |  | On-treatment^¶^ |  |  |  | 1 (0.6) |
|  |  | Post-treatment |  |  |  | 1 (0.6) |
|  | Skin cancer | On-treatment |  |  |  | 1 (0.6) |
|  | Squamous cell carcinoma^¶^ | Post-treatment |  |  |  | 1 (0.6) |
|  | Transitional cell carcinoma | Post-treatment |  |  |  | 1 (0.6) |

**SUPPLEMENTARY TABLE S12** Data displayed with descriptive statistics [frequencies (%)] for the SAF (n=168).

Abbreviations: MedDRA, Medical Dictionary for Regulatory Activities; PT, Preferred Term; SAF, Safety Analysis Set; SPM, Second Primary Malignancy.

^†^Patients with more than one second primary malignancies were counted only once in the category total (any event).

Three patients were reported with two documented second primary malignancies during or after lenalidomide treatment (corresponding patient-IDs were redacted due to data protection rules):

^‡^Patient 1: (1) Myelodysplastic syndrome (MedDRA PT) / (2) Acute myeloid leukaemia (MedDRA PT).

^§^Patient 2: (1) Bowen’s disease (MedDRA PT) / (2) Squamous cell carcinoma of skin (MedDRA PT).

^¶^Patient 3: (1) Prostate cancer (MedDRA PT) / (2) Squamous cell carcinoma (MedDRA PT).

^≠^Metastasis (PT): reported by the physician as “colon carcinoma with metastases in liver and lung” (MedDRA Reported Term).

^#^Prostate cancer (PT): reported by the physician as “progress of prostate carcinoma with infiltration of lower rectum” (MedDRA Reported Term).

1. **Subgroup analyses - G8-Geriatric assessment vs. age (>75 vs. ≤75 years)**

**Supplementary Table S13** Comparison of subgroup analyses stratified by G8-Geriatric assessment vs. age (>75 vs. ≤75 years) [FAS / SAF]

|  | G8-Geriatric  assessment tool^†^ | | |  | Age  (>75 vs. ≤75 years) | | | |
| --- | --- | --- | --- | --- | --- | --- | --- | --- |
|  | Rd  [FAS^‡^: N=164; SAF^§^ N=168] | | |  | Rd  [FAS^‡^: N=164; SAF^§^ N=168] | | | |
|  | Impaired | Non-impaired | Missing | |  | >75 years | | ≤75 years |
| Selected baseline patient demographic characteristics [FAS]^‡^ | | | | | | | | |
| Age (years) | |  |  | |  | |  | |
| Median | 78.8 | 76.4 | 77.5 | | 79.3 | | 71.8 | |
| Comorbidities [n, (%)] | |  |  | |  | |  | |
| 0/1 | 14 (10.5) | 11 (33.3) | 9 (20.0) | | 21 (18.1) | | 8 (16.7) | |
| ≥2 | 77 (89.5) | 22 (66.7) | 36 (80.0) | | 95 (81.9) | | 40 (83.3) | |
| ECOG PS [n, (%)] | |  |  | |  | |  | |
| 0/1 | 62 (72.1) | 33 (100.0) | 31 (68.9) | | 87 (75.0) | | 39 (81.3) | |
| ≥2 | 20 (23.2) | 0 (0) | 9 (20.0) | | 22 (19.0) | | 7 (14.6) | |
| Missing/unknown | 4 (4.7) | 0 (0) | 5 (11.1) | | 7 (6.0) | | 2 (4.2) | |
| CrCl (mL/min) [n, (%)] | |  |  | |  | |  | |
| ≥60 | 32 (37.2) | 22 (66.7) | 23 (48.9) | | 41 (35.3) | | 36 (75.0) | |
| <60 | 54 (62.8) | 11 (33.3) | 22 (51.1) | | 75 (64.7) | | 12 (25.0) | |
| ISS Stage [n, (%)] | |  |  | |  | |  | |
| I/II | 50 (58.1) | 23 (69.7) | 25 (55.6) | | 61 (52.6) | | 37 (77.1) | |
| III | 27 (31.4) | 8 (24.2) | 5 (11.1) | | 32 (27.6) | | 8 (16.7) | |
| Missing/unknown | 9 (10.5) | 2 (6.1) | 15 (33.3) | | 23 (19.8) | | 3 (6.3) | |
| Lenalidomide treatment characteristics [SAF]^§^ | |  |  | |  | |  | |
| Starting dose [n, (%)] | |  |  | |  | |  | |
| 25mg | 40 (44.9) | 23 (67.6) | 29 (64.4) | | 56 (48.3) | | 36 (69.2) | |
| <25mg | 49 (55.1) | 11 (32.4) | 16 (35.6) | | 60 (51.7) | | 16 (30.8) | |
| Duration (months) | |  |  | |  | |  | |
| Median (95% CI) | 6.8 (4.6, 8.1) | 9.9 (3.8, 22.0) | 9.8 (7.0, 14.2) | | 6.9 (5.0, 8.0) | | 14.9 (7.1, 24.9) | |
| Dose / day of administration^¶^ | |  |  | |  | |  | |
| Median (mg) | 15.3 | 20.7 | 17.7 | | 15.0 | | 18.9 | |
| Adverse events [SAF] ^§^ | |  |  | |  | |  | |
| Adverse events [n, (%)] | |  |  | |  | |  | |
| Grade 3/4 | 57 (64.0) | 20 (58.8) | 31 (68.9) | | 72 (62.1) | | 36 (69.2) | |
| SAE | 55 (61.8) | 14 (41.2) | 27 (60.0) | | 67 (57.8) | | 29 (55.8) | |
| Fatal outcome | 8 (9.0) | 2 (5.9) | 5 (11.1) | | 10 (8.6) | | 5 (9.6) | |
| Treatment-related AE [n, (%)] | | | | | | | | |
| Grade 3/4 | 25 (28.1) | 8 (23.5) | 12 (26.7) | | 27 (23.3) | | 18 (34.6) | |
| SAE | 12 (13.5) | 3 (8.8) | 8 (17.8) | | 15 (12.9) | | 8 (15.4) | |
| Treatment modification due to AE [n, (%)] | |  |  | |  | |  | |
| Interruption | 52 (58.4) | 17 (50.0) | 29 (64.4) | | 67 (57.8) | | 31 (59.6) | |
| Dose reduction | 23 (25.8) | 12 (35.3) | 17 (37.8) | | 32 (27.6) | | 20 (38.5) | |
| Discontinuation | 41 (46.1) | 14 (41.2) | 19 (42.2) | | 51 (44.0) | | 23 (44.2) | |
| Lenalidomide | | | | | | | | |
| Discontinuation due to toxicity | 27 (30.3) | 12 (35.3) | 11 (24.4) | | 35 (30.2) | | 15 (28.8) | |
| Effectiveness [FAS] ^‡^ | |  |  | |  | |  | |
| ORR | |  |  | |  | |  | |
| n, (%) | 46 (53.5) | 22 (66.7) | 29 (64.4) | | 63 (54.3) | | 34 (70.8) | |
| (95% CI) | (43.0, 63.7) | (49.5, 80.3) | (49.8, 76.8) | | (45.3, 63.1) | | (56.7, 81.8) | |
| PFS (months) | |  |  | |  | |  | |
| Median | 19.3 | 45.6 | 25.4 | | 19.3 | | 31.5 | |
| (95% CI) | (13.7, 25.2) | (19.3, 66.5) | (18.6, 33.8) | | (14.2, 25.0) | | (22.0, 48.0) | |
| OS (months) | |  |  | |  | |  | |
| Median | 44.1 | 84.6 | 65.8 | | 50.0 | | 83.7 | |
| (95% CI) | (27.2, 60.1) | (52.5, NA) | (31.8, NA) | | (33.7, 68.8) | | (45.7, NA) | |

**SUPPLEMENTARY TABLE S13** Data displayed with descriptive statistics [median (range) or frequencies (%)] (SAF; n = 168)

Abbreviations: AE, Adverse Event; CrCl, Creatinine Clearance; CI, Confidence Interval; ECOG PS, Eastern Cooperative Oncology Group Performance Status; G8-GA, G8-Geriatric assessment; ISS, International Scoring System; mL, Millilitre; Min, Minute; SAE, Serious Adverse Event; SAF, Safety Analysis Set.

^†^The validated G8-Geriatric assessment tool was used to assess the individual health status of the patient; impaired patient status is defined as G8-GA score ≤14, and non-impaired patient status is defined as G8-GA score >14, respectively).

^‡^FAS: Total number of patients: G8-GA impaired vs. non-impaired vs. missing: 86 vs. 33 vs. 45; patients >75 vs. ≤75 years: 116 vs. 48.

^§^SAF: Total number of patients G8-GA impaired vs. non-impaired vs. missing: 89 vs. 34 vs. 45; patients >75 vs. ≤75 years: 116 vs. 52. ^¶^Lenalidomide daily dose was defined as the cumulative dose divided by dose exposure (mg/day).

1. **References**

1. Benboubker L, Dimopoulos MA, Dispenzieri A, et al. Lenalidomide and dexamethasone in transplant-ineligible patients with myeloma. *N Engl J Med*. 2014;371(10):906-917. doi:10.1056/NEJMoa1402551

2. Facon T, Dimopoulos MA, Dispenzieri A, et al. Final analysis of survival outcomes in the phase 3 FIRST trial of up-front treatment for multiple myeloma. *Blood*. 2018;131(3):301-310. doi:10.1182/blood-2017-07-795047

3. Hulin C, Belch A, Shustik C, et al. Updated Outcomes and Impact of Age With Lenalidomide and Low-Dose Dexamethasone or Melphalan, Prednisone, and Thalidomide in the Randomized, Phase III FIRST Trial. *J Clin Oncol Off J Am Soc Clin Oncol*. Published online June 20, 2016. doi:10.1200/JCO.2016.66.7295

4. Bellera CA, Rainfray M, Mathoulin-Pélissier S, et al. Screening older cancer patients: first evaluation of the G-8 geriatric screening tool. *Ann Oncol Off J Eur Soc Med Oncol ESMO*. 2012;23(8):2166-2172. doi:10.1093/annonc/mdr587

5. Soubeyran P, Bellera C, Goyard J, et al. Screening for Vulnerability in Older Cancer Patients: The ONCODAGE Prospective Multicenter Cohort Study. *PLoS ONE*. 2014;9(12):e115060. doi:10.1371/journal.pone.0115060

6. Facon T, Dimopoulos MA, Meuleman N, et al. A simplified frailty scale predicts outcomes in transplant-ineligible patients with newly diagnosed multiple myeloma treated in the FIRST (MM-020) trial. *Leukemia*. 2020;34(1):224-233. doi:10.1038/s41375-019-0539-0

7. Delforge M, Minuk L, Eisenmann JC, et al. Health-related quality-of-life in patients with newly diagnosed multiple myeloma in the FIRST trial: lenalidomide plus low-dose dexamethasone versus melphalan, prednisone, thalidomide. *Haematologica*. 2015;100(6):826-833. doi:10.3324/haematol.2014.120121

8. Cocks K, King MT, Velikova G, et al. Evidence-based guidelines for interpreting change scores for the European Organisation for the Research and Treatment of Cancer Quality of Life Questionnaire Core 30. *Eur J Cancer*. 2012;48(11):1713-1721. doi:10.1016/j.ejca.2012.02.059

9. Sully K, Trigg A, Bonner N, et al. Estimation of minimally important differences and responder definitions for EORTC QLQ-MY20 scores in multiple myeloma patients. *Eur J Haematol*. 2019;103(5):500-509. doi:10.1111/ejh.13316
